# Ethanol-activated CaMKII signaling induces neuronal apoptosis through Drp1-mediated excessive mitochondrial fission and JNK1-dependent NLRP3 inflammasome activation

**DOI:** 10.1186/s12964-020-00572-3

**Published:** 2020-08-12

**Authors:** Jae Ryong Lim, Hyun Jik Lee, Young Hyun Jung, Jun Sung Kim, Chang Woo Chae, Seo Yihl Kim, Ho Jae Han

**Affiliations:** 1 Department of Veterinary Physiology, College of Veterinary Medicine, Research Institute for Veterinary Science, and BK21 PLUS Program for Creative Veterinary Science Research, Seoul National University, Seoul, 08826 Republic of Korea; 2 Laboratory of Veterinary Physiology, College of Veterinary Medicine, Chungbuk National University, Cheongju, Chungbuk 28644 South Korea; 3 Institute for Stem Cell & Regenerative Medicine (ISCRM), Chungbuk National University, Cheongju, Chungbuk 28644 South Korea

**Keywords:** Ethanol, NMDA receptor, NLRP3 inflammasome, CaMKII, JNK1, Drp1, Caspase-1, Mitophagy, Neuronal apoptosis

## Abstract

**Background:**

Neurodegeneration is a representative phenotype of patients with chronic alcoholism. Ethanol-induced calcium overload causes NOD-like receptor protein 3 (NLRP3) inflammasome formation and an imbalance in mitochondrial dynamics, closely associated with the pathogenesis of neurodegeneration. However, how calcium regulates this process in neuronal cells is poorly understood. Therefore, the present study investigated the detailed mechanism of calcium-regulated mitochondrial dynamics and NLRP3 inflammasome formation in neuronal cells by ethanol.

**Methods:**

In this study, we used the SK-N-MC human neuroblastoma cell line. To confirm the expression level of the mRNA and protein, real time quantitative PCR and western blot were performed. Co-immunoprecipitation and Immunofluorescence staining were conducted to confirm the complex formation or interaction of the proteins. Flow cytometry was used to analyze intracellular calcium, mitochondrial dysfunction and neuronal apoptosis.

**Results:**

Ethanol increased cleaved caspase-3 levels and mitochondrial reactive oxygen species (ROS) generation associated with neuronal apoptosis. In addition, ethanol increased protein kinase A (PKA) activation and cAMP-response-element-binding protein (CREB) phosphorylation, which increased N-methyl-D-aspartate receptor (NMDAR) expression. Ethanol-increased NMDAR induced intracellular calcium overload and calmodulin-dependent protein kinase II (CaMKII) activation leading to phosphorylation of dynamin-related protein 1 (Drp1) and c-Jun N-terminal protein kinase 1 (JNK1). Drp1 phosphorylation promoted Drp1 translocation to the mitochondria, resulting in excessive mitochondrial fission, mitochondrial ROS accumulation, and loss of mitochondrial membrane potential, which was recovered by Drp1 inhibitor pretreatment. Ethanol-induced JNK1 phosphorylation activated the NLRP3 inflammasome that induced caspase-1 dependent mitophagy inhibition, thereby exacerbating ROS accumulation and causing cell death. Suppressing caspase-1 induced mitophagy and reversed the ethanol-induced apoptosis in neuronal cells.

**Conclusions:**

Our results demonstrated that ethanol upregulated NMDAR-dependent CaMKII phosphorylation which is essential for Drp1-mediated excessive mitochondrial fission and the JNK1-induced NLRP3 inflammasome activation resulting in neuronal apoptosis.

Video abstract

**Graphical abstract:**

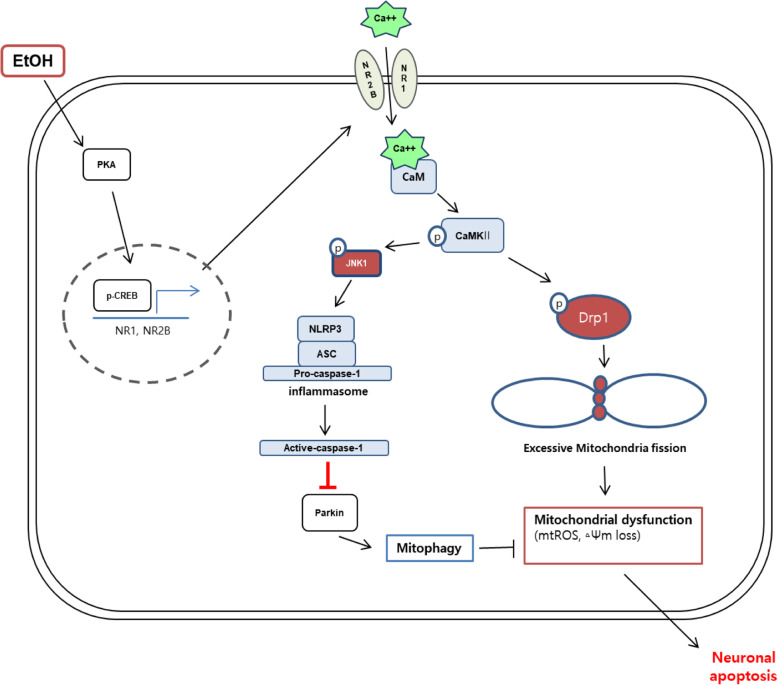

## Background

Heavy alcohol consumption is one of the risk factors of dementia, a common neurodegenerative disease characterized by neuronal cell death [1, 2]. Many studies have reported that ethanol induces cell death through increases in intracellular calcium concentrations [3–5]. In a previous study, an increase in excitatory amino acids, such as glutamate, which induces calcium-dependent excitotoxicity through N-methyl-D-aspartate receptor (NMDAR) overstimulation promoting neuronal cell death, was observed in patient with chronic alcoholism [6–8]. In addition, a recent study suggested that chronic ethanol treatment induced calcium overload by increasing NR1 expression and neuronal apoptosis [9]. Although calcium overload via NMDAR was found to play an important role in ethanol-induced neuronal apoptosis, the detailed mechanisms need to be investigated further.

Ethanol usually causes harmful effects on maintenance of mitochondria function which is important for cell survival [10–12]. Mitochondria are dynamic organelles and the balance of mitochondrial fission and fusion is important for the maintenance of mitochondrial function. A previous report indicated that an imbalance of mitochondrial dynamics, such as excessive mitochondrial fission, induced mitochondria damage leading to neuronal apoptosis [13]. Among the mitochondrial dynamin-related GTPases, dynamin-related protein 1 (Drp1) is the most notable protein and plays various roles in mitochondrial fission, mitophagy, bulk autophagy and neuronal cell death [14, 15]. A recent study reported that ethanol promoted mitochondrial reactive oxygen species (ROS) production and mitochondrial dysfunction through increases in Drp1-mediated mitochondrial fission [16]. In addition, some research has suggested that calcium signaling is a key regulator of Drp1-mediated mitochondrial fragmentation in neuronal cells [17–19]. However, the mechanism of mitochondrial fission and the effect of mitochondrial fission on apoptosis in neuronal cells exposed to ethanol are still unclear. Hence, the study on the mechanism of mitochondrial fission via calcium signaling will be help to understand ethanol-induced neuronal apoptosis.

Ethanol activates the NOD-like receptor protein 3 (NLRP3) inflammasome which induces programmed necrotic cell death [20, 21]. Many researchers have reported that intracellular calcium overload induces inflammasome formation and that calmodulin-dependent protein kinase II (CaMKII) is involved in the process of inflammasome activation [22–24]. In addition, recent studies have reported that NLRP3 phosphorylation by c-Jun N-terminal protein kinase 1 (JNK1) is essential for inflammasome activation [25, 26]. These results suggest that CaMKII may be involved in inflammasome activation as an upstream molecule of JNK1. Since no reports have investigated inflammasome activation via the CaMKII signaling pathway, a study of the relationship between CaMKII and JNK1 is meaningful. Inflammasomes have been found mainly in macrophages and microglia that release pro-inflammatory cytokines [27, 28]. Unlike previous reports, recent studies have shown that pro-inflammatory cytokines were released from neurons, although in lower amounts than glial cells [29–32]. In addition, one study has shown that ethanol-induced inflammasome formation in rat cortical neurons [33]. Moreover, the inhibition of caspase-1 activated by an inflammasome reduced apoptosis marker cleaved caspase-3 in primary cortical neurons [34]. Although several studies have reported that the inflammasome is involved in neuronal apoptosis, the direct and indirect mechanisms of inflammasome-mediated apoptosis have not been elucidated in neuronal cells exposed to ethanol. Recently, a study reported that inflammasome-induced caspase-1 activation inhibited mitophagy by cleaving parkin, a mitophagy regulator protein, and promoted cell death in macrophage cell lines [35]. These findings suggest that the ethanol-induced inflammasome can be a regulator of parkin-mediated mitophagy in neuronal cells. Since there are no reports on the mechanism of inflammasome involvement in neuronal apoptosis, identifying the relationship between inflammasomes and mitophagy will provide insights into the prevention of ethanol-induced neuronal apoptosis.

In this experiment, we used the SK-N-MC human neuroblastoma cell line to study the mechanism of ethanol-induced neuronal apoptosis. This neuroblastoma cell line has been used widely to investigate the pathogenesis of neurodegenerative diseases, including Alzheimer’s disease and other dementias. In addition, the neuroblastoma cell line has the advantages of high stability and reproducibility. Therefore we investigated the detailed mechanism of neuronal apoptosis through the calcium overload-mediated excessive mitochondrial fission and NLRP3 inflammasome activation in SK-N-MC exposed to ethanol.

## Methods

### Materials

The human neuroblastoma cell line SK-N-MC was obtained by Korean Cell Line Bank (Seoul, Korea). Fetal bovine serum (FBS) was purchased from Hyclone (Logan, UT, USA). Antibiotics and serum replacement (SR) were purchased from Gibco (Grand Island, NY, USA) The antibodies of β-actin, Cat-PKA, CREB, *p*-CREB (Ser113), p-CaMKII (Thr 286), CaMKII, CaM, p-JNK, ASC, BNIP3, NIX were purchased from Santa Cruz Biotechnology (Santa Cruz, CA, USA). The antibodies of Drp1, p-Drp1 (Ser 616), PINK1, Cleaved caspase-3 and Small interfering RNAs (siRNAs) for *JNK1* were purchased from Cell Signaling Technology, Inc. (Danvers, MA, USA). The antibodies of NR1, TOMM20, parkin and Na^+^/K^+^-ATPase were purchased from Abcam (Cambridge, England). The antibody of NR2B was purchased from Invitrogen Corporation (Camarillo, CA, USA). The antibodies of COX4, JNK1 (MAPK8) were purchased from CusaBio (Houston, TX, USA). The antibody of NLRP3 was purchased from AdipoGen Life Sciences. The antibodies of caspase-1, LC3 purchased from Novus Biologicals (Littleton, CO, USA). CM-H_2_DCFDA, MitoSOX™ Red, Mitotracker™ Green, Mitotracker™ Red were obtained from Thermo Fisher (Waltham, MA, USA). The 14–22 amide was obtained from Calbiochem (Merck Millipore). NAC, MitoTEMPO, SP600125, Ac-YVAD-cmk, Mdivi-1, KN-93, MK-801 were purchased from Sigma Chemical Company (St. Louis, MO, USA). Small interfering RNAs (siRNAs) for *CREB1*, *CASP1* and non-targeting (NT) were purchased from Dharmacon (Lafayette, CO, USA).

### Cell culture

The SK-N-MC cells were cultured in high-glucose Dulbecco’s Modified Eagle Medium (DMEM) supplemented with 10% FBS and 1% antibiotics. Cells were seeded in 60 or 100 mm diameter culture dishes, or in 6- or 12-well plates and incubated at 37 °C incubator with 5% CO_2_. When cells were grown 60–70% confluence, the medium was exchanged with serum-free medium containing 2% SR prior to experiments.

### Real time quantitative PCR

RNA was extracted from SK-N-MC using MiniBEST Universal RNA Extraction Kit (TaKaRa, Otsu, Shinga, Japan). Reverse transcription polymerase chain reaction (RT-PCR) was carried out using 1 μg of extracted RNA and a Maxime™ RT-PCR premix kit (iNtRON Biotechnology, Sungnam, Korea). RT-PCR was performed for 60 min at 45 °C to cDNA synthesis and 5 min RTase inactivation at 95 °C. The cDNA was amplified using Quanti NOVA SYBR Green PCR Kits (Qiagen, Hilden, Germany). Real-time quantification of RNA targets was carried out using RotorGene 6000 realtime thermal cycling system (Corbett Research, NSW, Australia) with mRNA primers and 1 μg of cDNA sample. Human primer sequences are described in Table S1. The Real-Time PCR was performed as follows: 15 min at 95 °C for DNA polymerase activation; 15 s at 95 °C for denaturing; and 40 cycles of 15 s at 94 °C, 30 s at 56 °C, and 30 s at 72 °C. Data were collected during the extension step (30 s at 72 °C), and analysis was performed with software provided by Rotor-Gene 6000 Series software (Qiagen, Hilden, Germany) to verify the specificity and identity of the PCR products.

### Western blot analysis

Cells were collected by using scraper after being washed once with cold PBS and incubated for 30 min on ice with RIPA buffer (ATTO Corporation, Tokyo, Japan) and a proteinase and phosphatase inhibitor (Thermo Fisher). The lysate were then cleared by centrifugation (15,000 rpm, 4 °C, 20 min). The Protein concentration was determined by BCA assay kit (Bio-Rad, Hercules, CA, USA). Samples containing 10 μg of protein were prepared for 6–15% sodium dodecyl sulfate polyacrylamide gel electrophoresis (SDS-PAGE) and then transferred to a polyvinylidene fluoride (PVDF) membrane. The membrane was blocked with 5% skim milk (Gibco) for 50 min and blocked membrane was washed with TBST solution 4 times every 8 min. After that, membrane was incubated with primary antibody overnight at 4 °C. The membrane was washed and incubated with HRP-conjugated secondary antibody (1:10,000) at room temperature for 2 h. The western blotting bands were visualized by using chemiluminescence (BioRad, Hercules, CA, USA). Densitometric analysis was performed with the Image J software (developed by Wayne Rasband, National Institutes of Health, Bethesda, MD, USA).

### Measurement of calcium

Fluo 3-AM was used to measure intracellular calcium levels. The cells on 6-well dishes washed with a PBS once and then incubated in PBS containing 2 μM Fluo 3-AM for 30 min at 37 °C in dark. Cells were treated with a 0.05% trypsin for 3 min and then centrifuged at 1500 g for 5 min. After centrifugation, cells were washed once with PBS, followed by suspending the cells in 400 μL PBS. Relative fluorescence intensity (RFI) of Fluo 3-AM was measured using flow cytometry (CytoFlex; Beckman Coulter, Fullerton, CA, USA).

### Measurement of intracellular reactive oxygen species levels

The cells were plated on 6- or 12-well dishes. Cells were washed once with PBS and incubated with 1 μM CM-H_2_DCFDA for 30 min at 37 °C in dark. Cells were treated with a 0.05% trypsin for 3 min and then centrifuged at 1,500 g for 5 min. Next, cells were washed once with PBS, followed by suspending the cells in 400 μL PBS. DCFDA staining was detected via flow cytometry (CytoFlex; Beckman Coulter, Fullerton, CA, USA).

### Measurement of mitochondrial ROS generation

The measurement of mitochondrial ROS generation was performed by using MitoSOX™ Red staining. Cells were washed once with PBS and incubated with 10 μM MitoSOX™ for 15 min at 37 °C in dark. Cells were then treated with a 0.05% trypsin for 3 min and then centrifuged at 1500 g for 5 min. Collected cells were suspended in 400 μL PBS. MitoSOX™-positive cells were detected by using flow cytometry (Beckman Coulter).

### Measurement of mitochondrial membrane potential and mitochondrial volume

To evaluate the mitochondrial membrane potential and volume, a TMRE (Sigma-Aldrich) and Mitotracker™ Green staining were used, respectively. After treatment, cells were incubated in 50 nM TMRE or 200 nM of Mitotracker™ for 20 min at 37 °C in dark. Cells were then treated with a 0.05% trypsin for 3 min and then centrifuged at 1500 g for 5 min. Collected cells were suspended in 400 μL PBS. Fluorescence intensities of TMRE or Mitotracker™ Green were detected by using flow cytometry.

### Annexin V/PI apoptosis detection

To measure apoptosis of cells, Annexin V and PI double staining was performed by using an annexin V/PI apoptosis detection kit (BD Bioscience, Franklin Lakes, NJ, USA) according to the supplier’s manual. Cells were detached with 0.05% trypsin and counted 1 × 10^5^ cells. Cells were then centrifuged at 1500 g for 5 min. Collected cells were suspended in the binding buffer supplied by the kit, and immunostained with AnnexinV-FITC (5 μL) and PI (5 μL) for 20 min at room temperature in dark. Cell apoptosis was measured by using flow cytometry (Beckman Coulter). Data were analyzed by using CytExpert software (Beckman Coulter). AnnexinV-positive and PI-negative (Q4), AnnexinV-positive and PI-positive (Q2), and AnnexinV-negative and PI-positive (Q1) were considered as early apoptotic, late apoptotic and necrotic cells, respectively. AnnexinV-negative and PI-negative (Q3) cells were considered viable. To measure the percentage of total apoptotic cells, the following formula was used: Apoptotic cells = Q2 + Q4.

### Immunocytochemistry

Cells were cultured on a confocal dish and fixed with 4% paraformaldehyde (Sigma Aldrich) for 10 min. 0.1% Triton X-100 was used for permeabilization for 5 min. To inhibit nonspecific binding of antibodies, cells were incubated with 1% normal goat serum for 30 min. Next cells were incubated with 1:100 dilution of primary antibody for 2 h at room temperature and washed with PBS three times. After washing, cells were incubated with Alexa Fluor 488 or 555-conjugated secondary antibody (1:300) in dark for 1 h in room temperature. Stained images were visualized by a super-resolution radial fluctuations (SRRF) imaging system (Andor Technology, Belfast, UK). Relative Fluorescence intensity was analyzed by using ImageJ software.

### Mitochondria morphology

The cells were plated on confocal dish and incubated with Mitotracker™ Red (200 nM) for 30 min at 37 °C. To visualize Mitotracker™ Red-stained cells, we used a super-resolution radial fluctuations (SRRF) imaging system. Analysis of images was performed by using FIJI software. Form factor (FF = perimeter^2^/4π • area) and aspect ratio (AR) were used for analysis of mitochondrial fragmentation [36, 37]. As both FF and AR approaches 1, they represent a circular shape, but the both parameters increase, the mitochondria morphology becomes elongated.

### Co-immunoprecipitation

Cells were lysed with the co-immunoprecipitation buffer (1% Triton X-100 in 50 mM Tris-HCl [pH 7.4] containing 150 mM NaCl, 5 mM EDTA, 2 mM Na_3_VO_4_, 2.5 mM Na_4_PO_7_, 100 mM NaF, 200 nM microcystin lysin-arginine, and protease inhibitor). Primary antibodies were immobilized with SureBeads™ Protein G magnetic beads (BioRad, Hercules, CA, USA, #161–4021). Immobilized magnetic beads were washed three times with PBST and then incubated with cell lysates (350 μg) for 12 h at 4 °C. Beads were washed three times with PBST and incubated with elution buffer (20 mM glycine pH 2.0) for 5 min. 1 M phosphate buffer and sample buffer were added to the samples.

### Small interfering RNA (siRNA) transfection

Prior to ethanol treatment, cells were incubated with 25 nM of the indicated siRNAs and transfection reagent TurboFect™ (Thermo Fisher, Waltham, MA, USA, #R0531) for 24 h in serum-free medium containing 2% SR. The non-targeting (NT) siRNA was used as the negative control and siRNAs sequences used for gene silencing are described in Table S2.

### Mitochondria fraction

Mitochondrial fraction was performed using mitochondria isolation kit according to the manufacturer’s instructions (Thermo Fisher Scientific). Cytoplasmic and mitochondrial proteins were extracted from cells according to the manufacturer’s instructions. Briefly, collected cells were incubated in Reagent A for 2 min on ice and then cell lysate was incubated with Reagent B for 5 min. Next, Reagent C was added to the cell lysate. After centrifugation, supernatant was used as a cytosolic fraction. The pellet was lysed with 2% CHAPS in Tris-buffered saline (25 mM Tris, 0.1 M NaCl, pH 7.2) solution and used as a mitochondrial fraction after centrifugation.

### Mitochondrial complex I activity

Prepare mitochondrial samples isolated from cells. Enzymatic activity of mitochondrial Complex I was measured according to the manufacturer’s instructions of the kit (Cat #:K968–100, BioVision, California, USA). Data was collected at 600 nm using a spectrophotometer and the kinetic reduction of complex I dye for 5 min was calculated as complex I activity.

### Measurement of cellular ATP levels

ATP Bioluminescent HSII kit (Roche) was used to measure intracellular ATP concentration according to the manufacturer’s instructions. ATP levels were detected with luminometer (Victor3; Beckman Coulter, Fullerton, CA, USA) and ATP concentrations were normalized to total protein concentration.

### Trypan blue exclusion cell viability assay

Cells were incubated with a 0.05% Trypsin to detach the cells. To identify dead cells, 0.4% trypan blue was added to the cell. Stained (dead) and unstained (live) cells were counted by using a Petroff–Hausser counting chamber (Hausser Scientific, Horsham, PA, USA). Cell viability = [{1− (number of trypan blue-stained cells/number of total cells)} × 100].

### Statistical analysis

Results are expressed as mean ± standard error of mean (S.E.M). Differential among experimental groups were analyzed by analysis of variance (ANOVA), and two group analysis was conducted by using Student’s *t* test. *P* value of < 0.05 was considered statistically significant.

## Results

### Role of ethanol-induced mitochondrial ROS accumulation in neuronal cell death

**Fig. 1:**
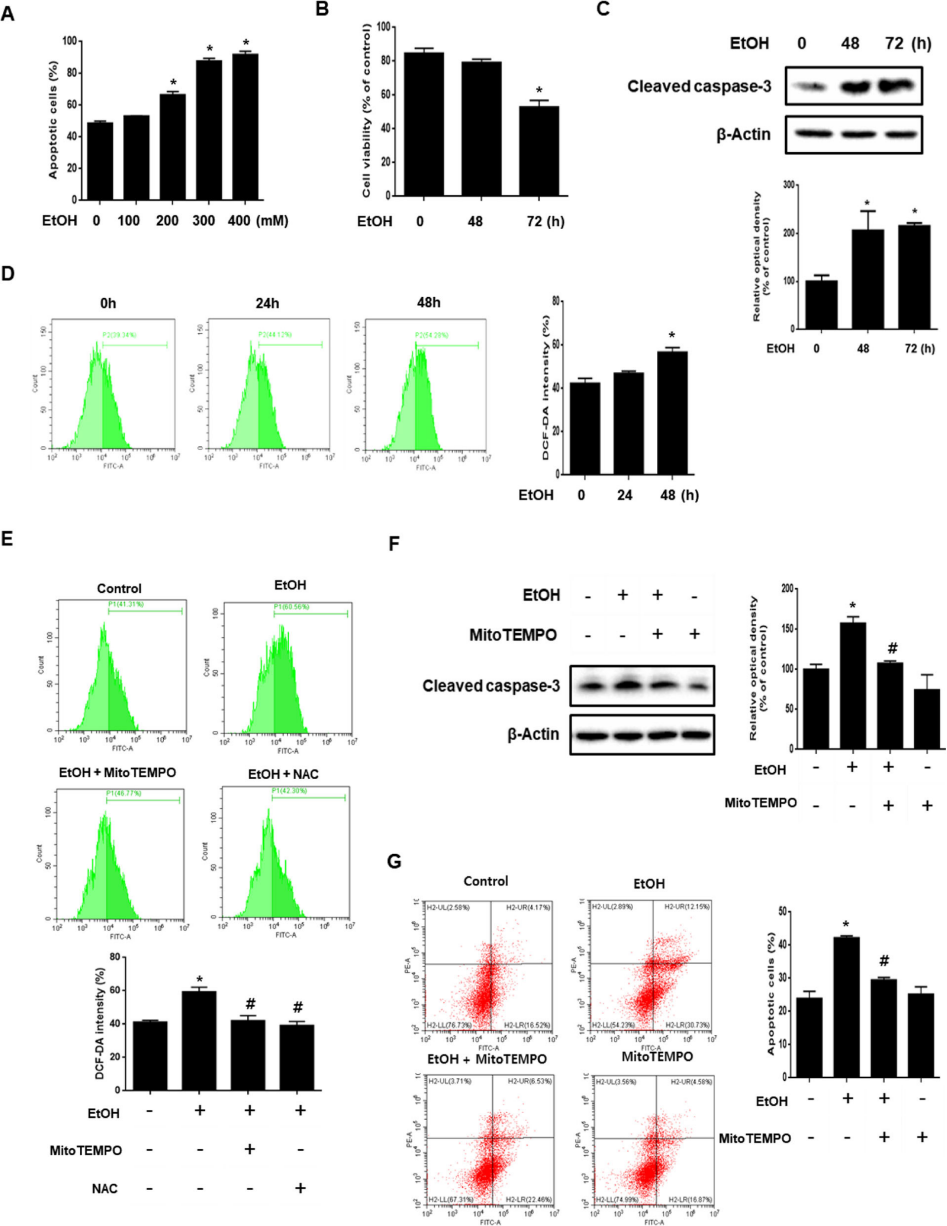
Role of ethanol-induced mitochondrial ROS accumulation in neuronal cell death. **a** SK-N-MC cells were incubated with various concentration of EtOH (100–400 mM) for 72 h. Quantitative analysis of the fold changes of late apoptotic cells were measured by using annexin V/PI staining with flow cytometry. Data are presented as a mean ± S.E.M. *n* = 4. **b** Cells were exposed to EtOH (200 mM) for 0–72 h. Cell viability was measured by trypan blue exclusion assay. Data are presented as a mean ± S.E.M. *n* = 4. **c** Cells were exposed to EtOH (200 mM) for 0–72 h. Cleaved caspase-3 was detected by western blot. Data are presented as a mean ± S.E.M. *n* = 3. **d** Cells were treated with EtOH (200 mM) in a time-dependent manner, ROS measurement by H_2_DCF-DA was conducted by flow cytometry. Data are presented as a mean ± S.E.M. *n* = 3. **e** Cells were pretreated with mitoTEMPO (2 μM) and NAC (5 mM) for 30 min and incubated with ETOH (200 mM) for 48 h. Then H_2_DCF-DA was incubated for 30 min to detect ROS and it was measured by using flow cytometry. Data are presented as a mean ± S.E.M. *n* = 3. **f** Cells were pretreated with mitoTEMPO (2 μM) for 30 min, and then exposed to EtOH (200 mM) for 48 h. Western blotting was conducted to determine the levels of cleaved caspase-3. Data are presented as a mean ± S.E.M. *n* = 4. **g** Cells were pretreated with mitoTEMPO (2 μM) for 30 min prior to EtOH treatment for 72 h. Apoptotic cells were detected by annexin V/ PI staining. Data are presented as a mean ± S.E.M. *n* = 3. All blot images are representative. *p < 0.05 versus control, ^#^*p* < 0.05 versus EtOH

We investigated the effect of various concentrations of ethanol (100–400 mM) on neuronal apoptosis. As shown in Fig. 1a, apoptosis occurred significantly in over 200 mM ethanol. Consistent with this result, the trypan blue assay results showed that ethanol reduced cell viability significantly at 72 h (Fig. 1b). In addition, ethanol-induced the cleavage of caspase-3 in a time dependent manner, suggesting that neuronal cell death induced by ethanol was related to apoptotic cell death (Fig. 1c). Next, we found that ethanol-induced ROS at 48 h (Fig. 1d). To confirm that the primary source of ROS induced by ethanol is mitochondria, we used the antioxidant MitoTEMPO, a mitochondria-specific superoxide scavenger, and general antioxidant NAC. The results showed that ROS generation induced by ethanol was significantly attenuated by the antioxidant (Fig. 1e). These results suggest that mitochondria are a major source of ROS induced by ethanol. To investigate the effect of mitochondrial ROS accumulation in neuronal cell death, we conducted experiments using MitoTEMPO. The western blot results showed a significant decrease in cleaved caspase-3 expression in the MitoTEMPO pretreated-cells compared to ethanol-treated cells without MitoTEMPO pretreatment (Fig. 1f). Moreover, the number of annexin V-positive cells was increased by ethanol and there were fewer annexin V-positive cells following MitoTEMPO pretreatment (Fig. 1g). Collectively, these results indicate that ethanol-induced mitochondrial ROS accumulation promoted neuronal apoptosis.

### Role of PKA/CREB pathway in ethanol-induced NMDA receptor expression and intracellular calcium overload

**Fig. 2:**
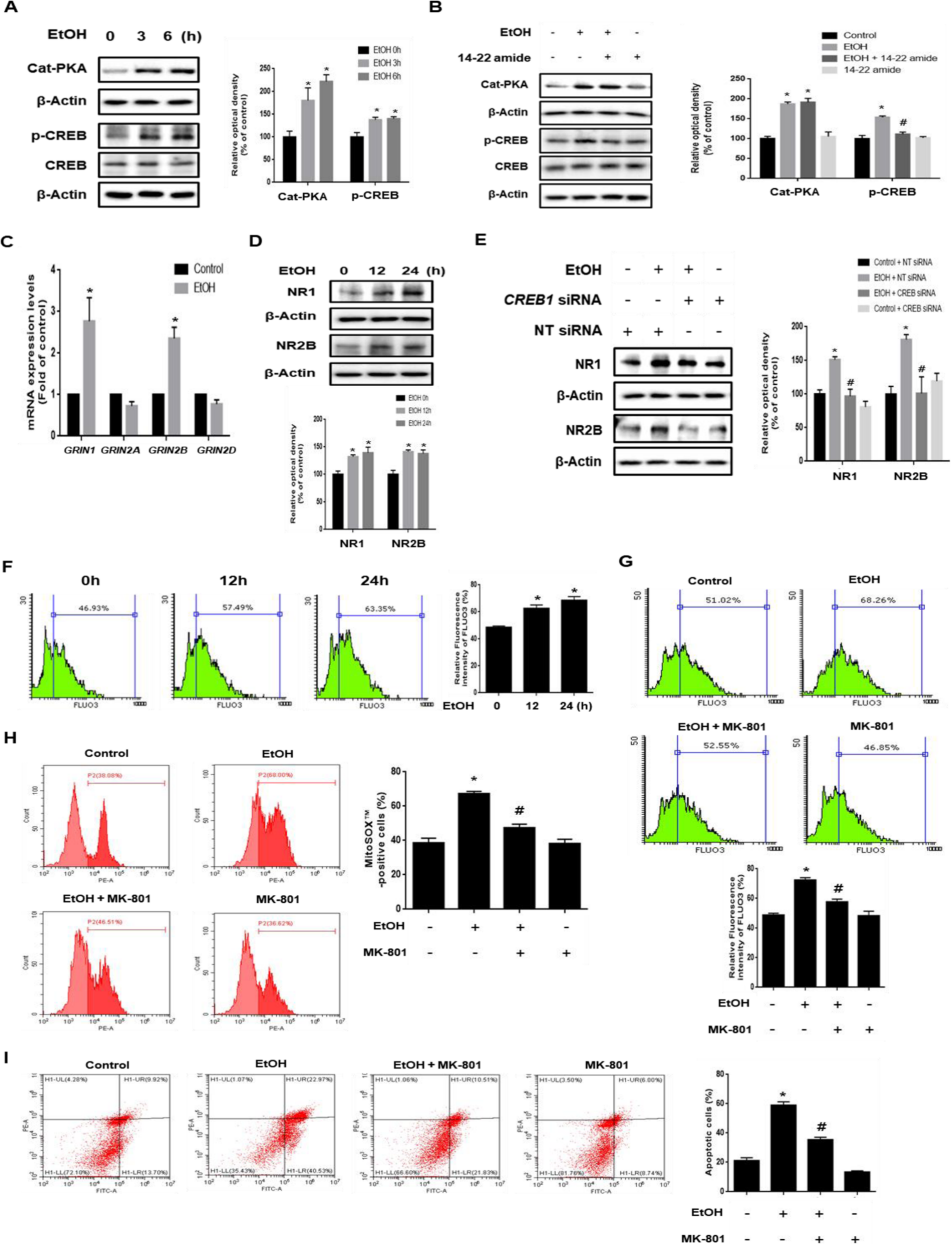
Role of PKA / CREB pathway in ethanol-induced NMDAR expression and intracellular calcium overload. **a** SK-N-MC cells were treated with EtOH (200 mM) for various time (0–6 h). Cat-PKA, p-CREB (Ser 113) and CREB were detected by western blot. β-Actin was used as a loading control. *n* = 3. **b** Cells were pretreated with 14–22 amide (1 μM) for 30 min before EtOH treatment. Cat-PKA, CREB and p-CREB (Ser 113) were analyzed by western blotting. β-Actin was used as a loading control. *n* = 4. **c** Cells were treated with EtOH (200 mM) for 12 h. mRNA expressions of *GRIN1*, *GRIN2A*, *GRIN2B*, and *GRIN2D* were analyzed by quantitative real time PCR. Data were normalized by the ACTB mRNA expression level. *n* = 4. **d** Cells were exposed to EtOH (200 mM) for 0–24 h. NR1 and NR2B were detected by western blot. *n* = 4. **e** Cells were transfected with *CREB* siRNA or NT siRNA for 24 h prior to ethanol exposure for 12 h. NR1 and NR2B expressions were measured by western blotting. *n* = 3. *p < 0.05 versus control with NT siRNA transfection, #*p* < 0.05 versus EtOH with NT siRNA transfection. **f** Cells were exposed to EtOH for 0–24 h and then loaded with Fluo 3-AM (2 μM) for 30 min. The amount of intracellular calcium was measured by using flow cytometry. *n* = 3**. g** Cells were pretreated with MK-801 (10 μM) for 30 min prior to EtOH treatment, subsequently loaded with Fluo 3-AM (2 μM) for 30 min. *n* = 4. **h** The population of MitoSOX™-positive cells was measured by flow cytometry. *n* = 3.** i** Apoptotic cells were measured by annexin V/PI analysis assay. All data are presented as a mean ± S.E.M. *n* = 3**.** All blot images are representative. *p < 0.05 versus control, ^#^*p* < 0.05 versus EtOH

We investigated the role of the PKA/CREB pathway in ethanol-increased NMDAR expression. As shown in Fig. 2a, ethanol increased catalytic PKA and the phosphorylation of CREB in a time-dependent manner. In addition, ethanol-induced catalytic PKA expression was not reduced by 14–22 amide (PKA inhibitor), but the CREB phosphorylation increased by ethanol was reduced by 14–22 amide pretreatment (Fig. 2b). These results indicate that 14–22 amide pretreatment inhibits the ethanol-induced CREB phosphorylation through regulation of PKA activity. To confirm the effect of ethanol on NMDAR expression, we performed real-time PCR and western blot. We observed that ethanol significantly stimulated *GRIN1* and *GRIN2B* mRNA expression (Fig. 2c). Consistent with this result, ethanol increased NR1 and NR2B protein expression in a time-dependent manner (Fig. 2d). In addition, the immunofluorescence results showed that ethanol increased expression and membrane accumulation of NR1 (Additional file 1). Based on the result that CREB acted as an NR1 and NR2B transcription factor, we investigated the role of CREB in NMDAR expression. As shown in Fig. 2e, ethanol significantly increased NR1 and NR2B expression, which were prevented by CREB silencing. Next, we investigated the role of the NMDAR in ethanol-induced calcium overload. Ethanol treatment elevated calcium influx into the cells in a time-dependent manner and the amount of intracellular calcium increased by ethanol treatment was decreased by MK-801 (NMDAR antagonist) pretreatment (Fig. 2f and g). However, ER stress inhibition did not identify any significant changes of ethanol-increased intracellular calcium levels (Additional file 2). To determine the role of the NMDAR in ethanol-induced mitochondrial ROS accumulation and neuronal apoptosis, we performed MitoSOX™ and annexin V/PI staining and measured the results by using flow cytometry. As shown in Fig. 2h and i, MK-801 pretreatment decreased the number of MitoSOX™-positive cells and the number of annexin V-positive cells. Taken together, these results suggest that ethanol-induced NMDAR expression via PKA/CREB pathway is critical for calcium overload, mitochondrial ROS accumulation, and neuronal apoptosis.

### Role of CaMKII / Drp1 pathway in ethanol-induced excessive mitochondrial fragmentation leading to mitochondrial dysfunction

**Fig. 3:**
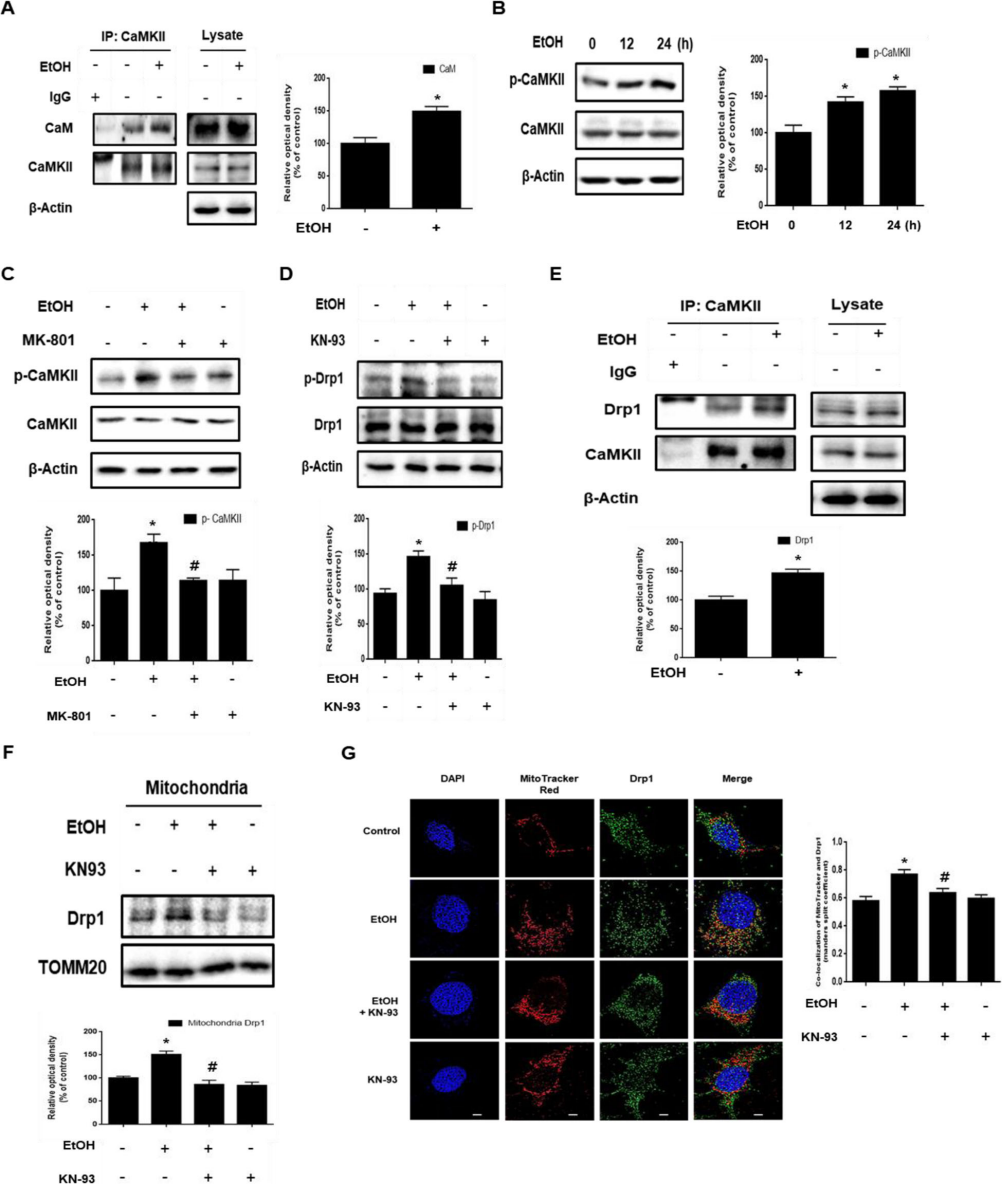
Ethanol-induced CaMKII activation promotes translocation of Drp1 to the mitochondria. **a** SK-N-MC cells were incubated EtOH (200 mM) for 12 h and then harvested. CaMKII was immunoprecipitated with an anti-CaM, anti-CaMKII antibodies (left). The expression of CaM, CaMKII, and β-Actin in total cell lysates is shown (right). *n* = 3**. b** Cells were treated with EtOH for various times (0–24 h). CaMKII and p-CaMKII (Thr 286) were analyzed by western blot. β-Actin was used as a loading control. *n* = 4. **c** Cells were pretreated with MK-801 (10 μM) for 30 min prior to EtOH treatment for 24 h. p-CaMKII (Thr 286) and CaMKII were detected by western blot. *n* = 4. **d** Cells were pretreated with KN-93 (1 μM) for 30 min prior to EtOH treatment for 24 h. Drp1 and p-Drp1 (Ser616) were detected by western blot. β-Actin was used as a loading control. *n* = 4. **e** Cells were incubated EtOH (200 mM) for 24 h and then harvested. CaMKII was immunoprecipitated with anti-CaMKII and anti-Drp1 antibodies (left). The expression of CaMKII, Drp1 and β-Actin in total cell lysates is shown (right). *n* = 3. **f** Cells were pretreated with KN-93 (1 μM) for 30 min prior to EtOH treatment for 48 h. Mitochondrial and cytosolic fractions were mentioned in methods. Drp1 was detected by western blot. Anti-TOMM20 was used as mitochondria marker. *n* = 3. **g** Cells were pre-treated with KN-93 (1 μM) for 30 min prior to EtOH treatment for 24 h and immunostained with Drp1 antibody and Mitotracker™. Co-localization of Drp1 (green) and Mitotracker™ (red) was visualized with SRRF imaging system. Scale bars are 8 μm (magnification, × 1,000). *n* = 4. All data are presented as a mean ± S.E.M. All blot and immunofluorescence images shown are representative. *p < 0.05 versus control, ^#^*p* < 0.05 versus EtOH.

**Fig. 4:**
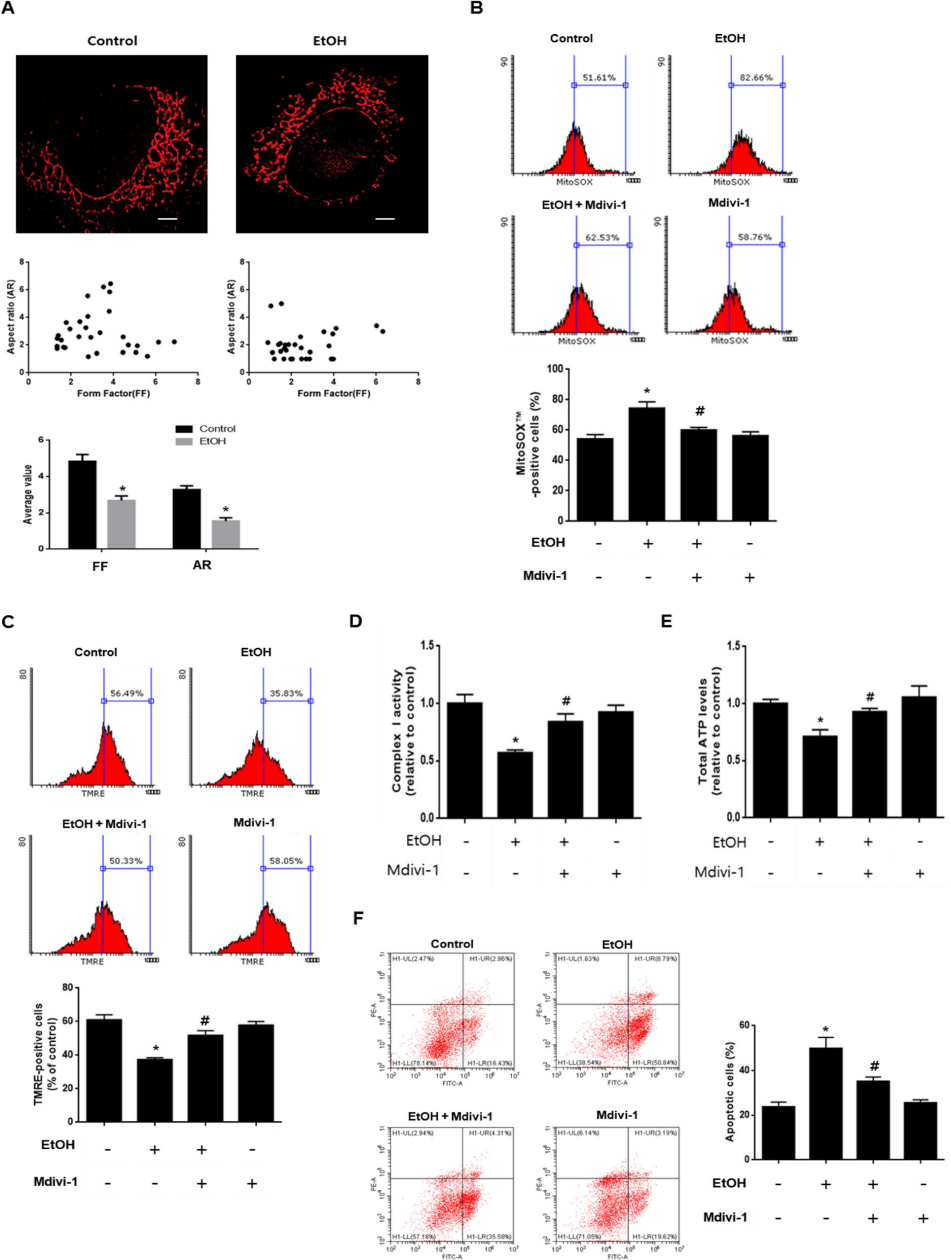
Ethanol-induced excessive mitochondrial fission leads to mitochondrial dysfunction. **a** SK-N-MC cells were exposed to EtOH for 48 h and then loaded with Mitotracker™ Red (200 nM). Representative images were visualized with SRRF imaging system. Scattered plots and average values for form factor (FF) and aspect ratio (AR) were shown on below. Data are presented as a mean ± S.E.M. *n* = 10. Scale bars are 8 μm (magnification, × 1,000). **b** Cells were pretreated with Mdivi-1 (1 μM) for 30 min before EtOH treatment for 48 h. Cells were stained with MitoSOX™. The population of MitoSOX™-positive cells was measured by flow cytometry. Data are presented as a mean ± S.E.M. *n* = 3.** c** Cells were stained with tetramethylrhodamine ethyl ester (TMRE). The population of TMRE-positive cells was measured by flow cytometry. Data are presented as a mean ± S.E.M. *n* = 3**. d, e** Cells were pretreated with Mdivi-1 (1 μM) for 30 min prior to EtOH treatment for 48 h. Mitochondrial complex I activity and intracellular ATP levels were measured respectively and then normalized to cellular protein. Data are presented as a mean ± S.E.M. *n* = 3. **f** Cells were pretreated with Mdivi-1 (1 μM) for 30 min before EtOH treatment for 72 h. Apoptotic cells were measured by annexin V/PI analysis assay. Data are presented as a mean ± S.E.M. *n* = 3**.** All blot and immunofluorescence images shown are representative. *p < 0.05 versus control, ^#^*p* < 0.05 versus EtOH

We investigated the mechanism involved in the ethanol-induced excessive mitochondrial fission in neuronal cells. The intracellular calcium level increased by ethanol promoted the affinity of the calmodulin (CaM) and CaMKII interactions (Fig. 3a). This binding increased CaMKII activity via autophosphorylation and the phosphorylation of CaMKII increased in a time-dependent manner (Fig. 3b). Moreover, CaMKII phosphorylation was blocked by MK-801, suggesting that ethanol-induced calcium overload regulates CaMKII activity (Fig. 3c). To examine whether CaMKII activation enhanced by ethanol affects Drp1 phosphorylation, the CaMKII inhibitor, KN-93, was used. Ethanol increased the phosphorylation of Drp1, which was attenuated by KN-93 (Fig. 3d). The binding interaction between CaMKII and Drp1 was significantly increased in cells treated with ethanol (Fig. 3e). These results suggest that CaMKII activated through ethanol-induced calcium overload binds directly to Drp1, facilitating increased phosphorylation of the Ser 616 residue. In addition, western blot and immunofluorescence results showed that the translocation of Drp1 to the mitochondria was increased by ethanol treatment, whereas it was blocked by KN-93 (Fig. 3f and g). Increased mitochondrial translocation of Drp1 by ethanol causes excessive mitochondrial fragmentation. We confirmed ethanol-induced excessive mitochondrial fragmentation using Mitotracker™ Red, a mitochondria specific fluorescent dye. Furthermore, the effects of ethanol on mitochondrial morphology were analyzed with the AR and FF. The AR and FF value decreased when treated with ethanol for 48 h, suggesting the fragmentation of mitochondria (Fig. 4a). Next, we investigated the effect of ethanol-induced excessive mitochondrial fission on mitochondrial dysfunction and neuronal cell death. To confirm the effect of excessive mitochondrial fission on mitochondrial ROS accumulation and mitochondrial membrane potential, we undertook MitoSOX™ and TMRE staining and measured the results by using flow cytometry. As shown in Fig. 4b, the inhibition of mitochondrial fission by Mdivi-1 decreased the number of MitoSOX™-positive cells, showing that mitochondrial fission plays a critical role in mitochondrial ROS generation. In addition, ethanol decreased the number of TMRE-positive cells, and the effect was prevented by pretreatment with Mdivi-1 (Fig. 4c). Moreover, mitochondrial complex I activity and total ATP level reduced by ethanol were recovered by Mdivi-1 (Fig. 4d and e). To examine whether ethanol-increased mitochondrial fission affects neuronal apoptosis, we used annexin V/PI staining. The number of annexin V-positive cells increased by ethanol was lower in Mdivi-1-pretreated cells (Fig. 4f). These results indicate that ethanol-induced excessive mitochondrial fission stimulated mitochondrial dysfunction and neuronal apoptosis.

### Role of CaMKII / JNK1 pathway in ethanol-induced NLRP3 inflammasome formation

**Fig. 5:**
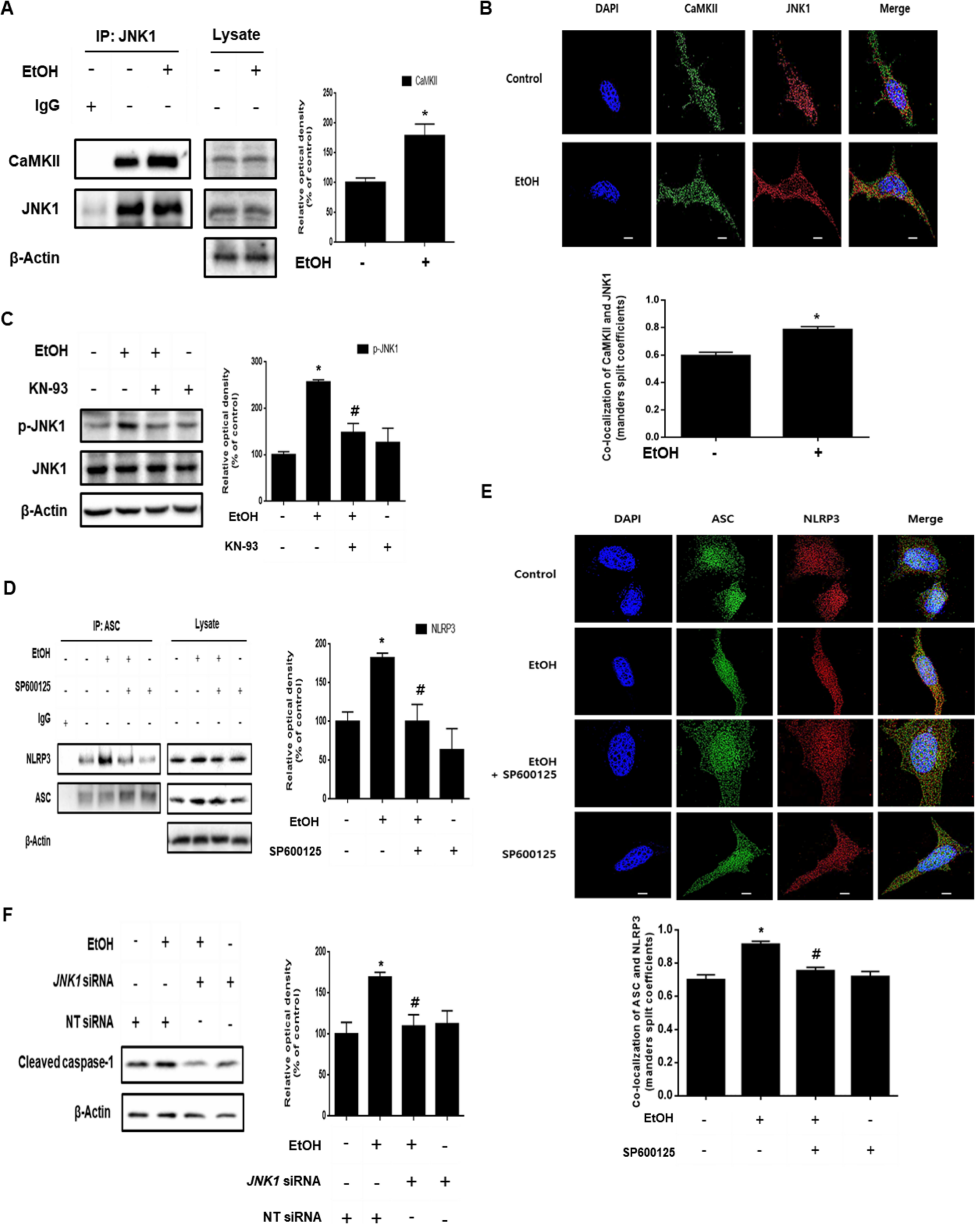
Role of CaMKII/JNK1 pathway in ethanol-induced inflammasome activation. **a** SK-N-MC cells were incubated EtOH (200 mM) for 24 h and then harvested. JNK1 was immunoprecipitated with anti-JNK1 and anti-CaMKII antibodies (left). The expression of JNK1, CaMKII, and β-Actin in total cell lysates is shown (right). Data are presented as a mean ± S.E.M. *n* = 3.** b** Cells were incubated with EtOH for 24 h and immunostained with CaMKII and JNK1 antibodies. Co-localization of CaMKII (green) and JNK1 (red) was visualized with SRRF imaging system. Scale bars are 8 μm (magnification, × 1,000). *n* = 5.** c** Cells were pre-treated with KN-93 (1 μM) for 30 min prior to EtOH treatment for 24 h. JNK1 and phosphorylated JNK1 (Thr 183/ Tyr 185) were analyzed by western blotting. β-Actin was used as a loading control. Data are presented as a mean ± S.E.M. *n* = 4. **d** Cells were pre-treated with SP600125 (10 μM) for 30 min prior to EtOH treatment for 48 h. ASC was immunoprecipitated with anti-ASC and anti-NLRP3 antibodies (left). The expression of ASC, NLRP3 and β-Actin in total cell lysates is shown (right). Data are presented as a mean ± S.E.M. *n* = 3.** e** Cells were immunostained with ASC and NLRP3 antibodies. Co-localization of ASC (green) and NLRP3 (red) was visualized with SRRF imaging system. Scale bars are 8 μm (magnification, × 1,000). *n* = 4.** f** Cells were transfected with *JNK1* siRNA or NT siRNA for 24 h prior to ethanol exposure for 48 h. Cleaved caspase-1 expression was measured by western blotting. β-Actin was used as a loading control. Data are presented as a mean ± S.E.M. *n* = 3. All blot and immunofluorescence images shown are representative. *p < 0.05 versus control, ^#^*p* < 0.05 versus EtOH.

Despite the fact that CaMKII is involved in inflammasome formation, detailed mechanisms have not been studied. Therefore, we investigated the mechanism of inflammasome formation through ethanol–induced CaMKII activation. To determine whether CaMKII and JNK1 interact with each other, we confirmed the binding between CaMKII and JNK1. As shown in Fig. 5a, the interaction between CaMKII and JNK1 was increased by ethanol treatment. In addition, the immunofluorescence results showed that ethanol increased the co-localization of the two proteins (Fig. 5b). We also pretreated the cells with KN-93 to determine the role of CaMKII in ethanol-induced JNK1 phosphorylation. As shown in Fig. 5c, we identified that pretreatment with KN-93 reduced the phosphorylation of JNK1. These results suggest that CaMKII is involved in JNK1 activation. Based on a recent study that reported NLRP3 phosphorylation by JNK1 was essential for the activation of the inflammasome [25], we investigated the role of JNK1 on inflammasome formation in neuronal cells exposed to ethanol. The co-immunoprecipitation and immunofluorescence results showed that ethanol significantly increased the co-localization of ASC and NLRP3, whereas the interaction was suppressed in cells treated with the JNK inhibitor SP600125 (Fig. 5d and e). Further, we confirmed that the cleaved caspase-1 expression in *JNK1* siRNA-transfected cells treated with ethanol was lower than that in NT siRNA-transfected cells treated with ethanol (Fig. 5f). These results demonstrate that ethanol-induced CaMKII signaling was critical for JNK1-mediated inflammasome formation.

### Role of ethanol-induced caspase-1 activation in parkin-mediated mitophagy inhibition

**Fig. 6:**
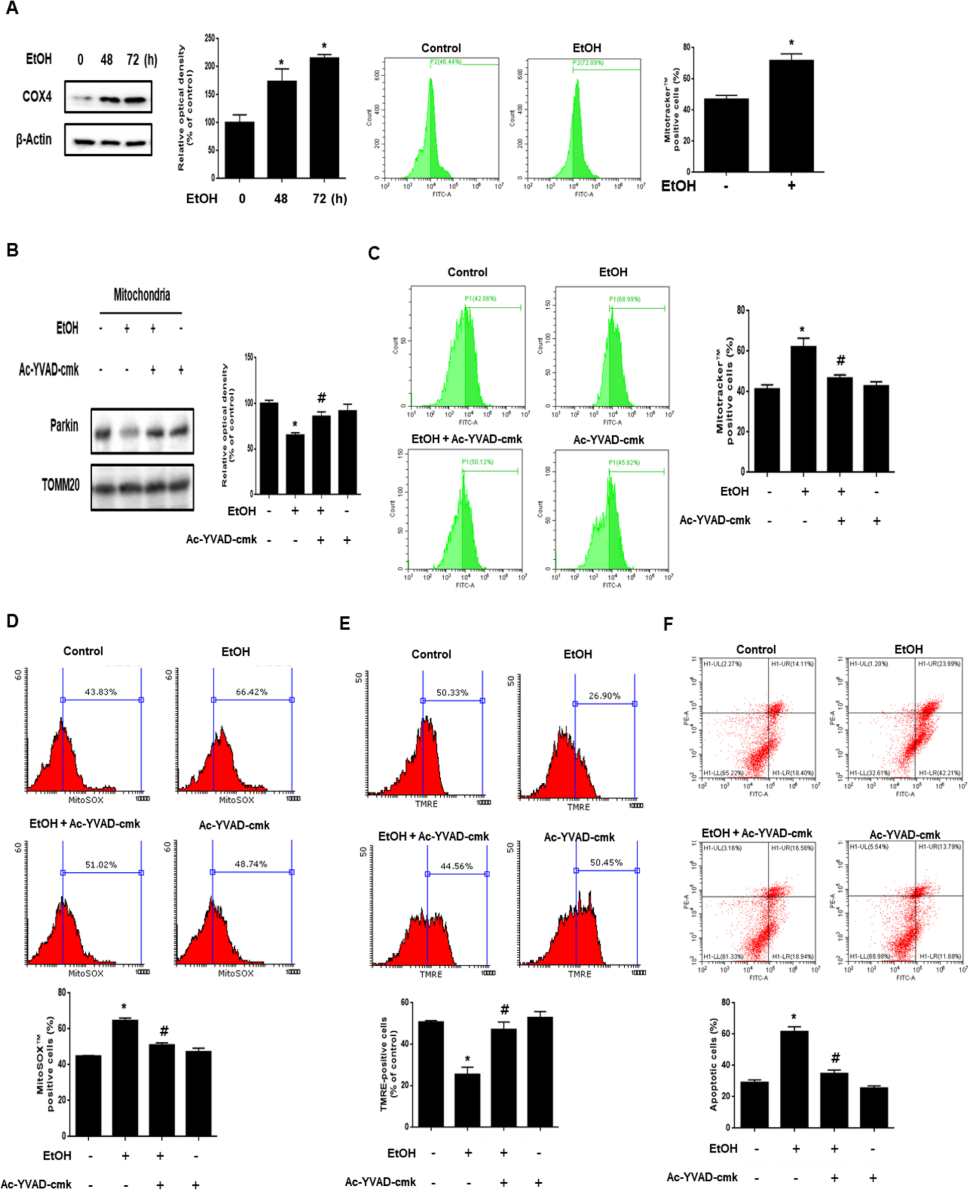
Role of ethanol-induced caspase-1 activation in parkin-mediated mitophagy inhibition. **a** Cells were exposed to EtOH (200 mM) for 0–72 h. COX4 was detected by western blot. β-Actin was used as a loading control. The population of Mitotracker™-positive cells was measured by flow cytometry. Data are presented as a mean ± S.E.M. *n* = 3. **b** parkin and TOMM20 with mitochondrial fractionized samples were detected by western blot. Data are presented as a mean ± S.E.M. *n* = 4. **c** Cells were pre-treated with Ac-YVAD-cmk (10 μM) for 30 min prior to EtOH treatment for 48 h. Cells were stained with Mitotracker™. The population of Mitotracker™-positive cells was measured by flow cytometry. Data are presented as a mean ± S.E.M. *n* = 4. **d** The population of MitoSOX™-positive cells was measured by flow cytometry. Data are presented as a mean ± S.E.M. *n* = 3. **e** The population of TMRE-positive cells was measured by flow cytometry. Data are presented as a mean ± S.E.M. *n* = 4.** f** Cells were pretreated with Ac-YVAD-cmk (10 μM) for 30 min before ethanol treatment for 72 h. Apoptotic cells were measured by annexin V/PI analysis assay. Data are presented as a mean ± S.E.M. *n* = 3**.** All blot images are representative. *p < 0.05 versus control, ^#^*p* < 0.05 versus EtOH

In the present study, we analyzed the mRNA and protein expression of mitophagy regulator genes, such as *PINK1, BNIP3*, and *NIX*, to determine the effect of ethanol on the expression of mitophagy regulators. As shown in the Additional file 3, ethanol increased *PINK1* mRNA and protein expression, but not those of BNIP3 or NIX. These results suggest that ethanol induces mitophagy through the PINK1/parkin pathway. To determine the effect of ethanol on mitophagy, we measured COX4 protein expression and Mitotracker™-positive cells via western blot and flow cytometry analysis, respectively. As shown in Fig. 6a, ethanol increased COX4 protein expression and the number of Mitotracker™-positive cells. These results suggest that ethanol inhibits mitophagy. A recent study reported that inflammasome-activated caspase-1 inhibits mitophagy by cleaving the parkin in a macrophage cell line [35]. Based on this finding, we hypothesized that activated caspase-1 would be involved in parkin translocation to the mitochondria in neuronal cells exposed to ethanol. Therefore, we investigated the effect of ethanol-activated caspase-1 in parkin translocation to the mitochondria in neuronal cells. The result showed that ethanol decreased parkin expression in the mitochondrial fraction, but this effect abolished by Ac-YVAD-cmk, caspase-1 inhibitor (Fig. 6b). In addition, the translocation of LC3-II to the mitochondria was also increased by *CASP1* siRNA transfection (Additional file 4). These results suggest that caspase-1 activated by ethanol bound to parkin and inhibited the translocation of parkin and LC3-II to the mitochondria. Subsequently, we performed flow cytometry analysis with Mitotracker™ to determine the role of caspase-1 activation in mitophagy. The number of Mitotracker™-positive cells in Ac-YVAD-cmk-treated cells was lower than that in ethanol-treated cells (Fig. 6c). We also measured mitochondrial ROS and mitochondrial membrane potential using MitoSOX™ and TMRE staining. The flow cytometry results of MitoSOX™ and TMRE showed that the increase in MitoSOX™-positive cells and the decrease in TMRE-positive cells by ethanol were prevented by suppressing caspase-1 (Fig. 6d and e). In addition, we investigated the effect of caspase-1 activation on neuronal cell apoptosis. We performed annexin V and PI double staining flow cytometry analysis with cells treated with ethanol for 72 h. Our results showed that the number of annexin V-positive cells in caspase-1-suppressed cells exposed to ethanol was lower than that of ethanol-treated non suppressed cells (Fig. 6f). To determine the effect of ethanol-induced mitophagy inhibition on neuronal cell death, we conducted experiments using trehalose, a mitophagy inducer. The results showed that the number of annexin V-positive cells increased by ethanol treatment was lower in cells pretreated with trehalose (Additional file 5). These results suggest that caspase-1 activated by ethanol-induced inflammasome formation inhibits mitophagy and exacerbates mitochondrial dysfunction causing neuronal apoptosis.

## Discussion

**Fig. 7:**
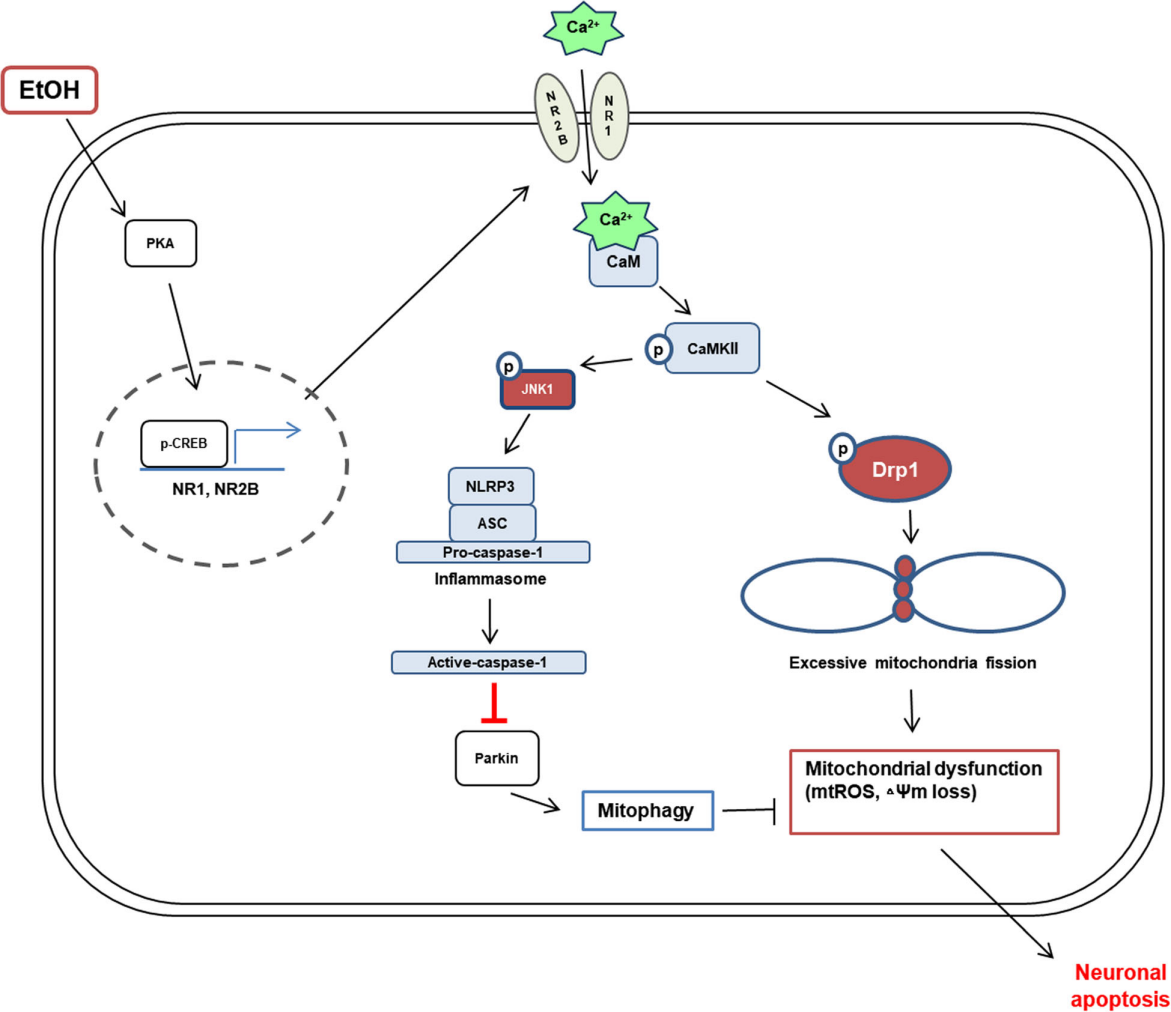
The schematic model for mechanism involved in EtOH-induced neuronal apoptosis. EtOH-induced NLRP3 inflammasome activation inhibits the mitophagy, which increases the accumulation of mitochondrial ROS by excessive fission leading to neuronal apoptosis

The results of this study elucidated the detailed mechanism by which ethanol-induced calcium signaling induces neuronal apoptosis (Fig. 7). An increase in excitatory amino acids has been reported in patients with chronic alcoholism, which promotes NMDAR-mediated excessive calcium influx leading to cell death [38]. A recent study showed that ethanol increases intracellular calcium than in a normal state through the upregulation of NMDAR expression and induces neuronal apoptosis [9]. In addition to many in vivo and in vitro studies, previous study has reported that chronic ethanol exposure increased the expression of NMDAR subunit genes in human embryonic stem cell-derived cortical neurons [39–42]. Consistent with these findings, we hypothesized that the main reason of calcium overload caused by ethanol is NMDAR. In present study, we confirmed that the ethanol-induced calcium overload was NMDAR-dependent. Furthermore, we demonstrated that ethanol significantly increased mRNA expression of *GRIN1* and *GRIN2B* more than other subunits of the NMDAR. These findings suggest that the NMDAR, particularly increased NR1 and NR2B expression, is the reason for the ethanol-induced intracellular calcium overload. In addition, this result is consistent with previous reports showing that an increase in NMDAR containing the NR2B subunit was associated with cell death mechanisms [43–45]. Previous studies have suggested that chronic ethanol treatment increased the PKA-dependent CREB phosphorylation [46] and that the expression of NR1 and NR2B was regulated by CREB in mice [47]. Consistent with a previous study, we confirmed that NR1 and NR2B protein expression decreased when CREB was inhibited, suggesting that CREB acts as an NMDAR transcription factor in neuronal cells. Taken together, we proposed that the ethanol-activated PKA/CREB pathway increases expression of NR1 and NR2B, which is a key factor in ethanol-induced calcium overload, and the regulation of the calcium influx via the NMDAR is a promising strategy to prevent neuronal apoptosis by ethanol.

The present work showed that ethanol-induced calcium overload promoted excessive mitochondrial fragmentation via CaMKII/Drp1 pathway. Many investigators have reported that excessive mitochondrial fragmentation was involved in the pathogenesis of neurodegenerative diseases. Although ethanol exposure has been reported to induce mitochondrial fragmentation [48], the effects and mechanism of ethanol-induced mitochondrial fragmentation in neuronal cells have not been investigated. Previous reports have shown that ethanol-induced mitochondrial fission promoted mitochondrial ROS generation leading to mitochondrial dysfunction in human retinal pigment epithelial cell lines [16]. The exact mechanism has not been explained, but Kitagaki et al. reported that ethanol-induced apoptosis was mediated by the mitochondrial fragmentation pathway in yeast [49]. In the present study, we confirmed that excessive mitochondrial fission-induced mitochondrial ROS generation and dysfunction were decreased by Drp1 inhibition in neuronal cells exposed to ethanol. Furthermore, Drp1 inhibition reduced ethanol-induced neuronal apoptosis. These results suggest that Drp1 acts as a regulator of ethanol-induced mitochondrial fission and that the regulation of Drp1-mediated mitochondrial fission can be a therapeutic target in neuronal apoptosis. Drp1 is a cytosolic protein whose activation and inactivation are regulated by the phosphorylation of specific residues [50, 51]. The phosphorylation of Drp1 at Ser 637 residue reduced the activation, whereas the phosphorylation of Drp1 at Ser 616 residue increased the activation and promoted the translocation of Drp1 to the mitochondria [52]. Previous studies have shown that CaMKII activated Drp1 via phosphorylation at Ser 616 and an increase in Drp1 translocation to the mitochondria induced excessive mitochondrial fragmentation [53, 54]. Our data showed that CaMKII activated by ethanol-induced calcium overload directly phosphorylated Drp1 at Ser 616 residue. This finding indicates that ethanol-activated CaMKII regulates Drp1 translocation from the cytosol to the mitochondria as an upstream molecule of Drp1.

Many studies have reported that an increase in intracellular calcium induces inflammasome formation and CaMKII signaling triggers inflammasome activation in cardiomyocytes [55–57]. These results suggest that CaMKII signaling is important for inflammasome activation. However, the detailed mechanism of CaMKII-mediated inflammasome formation has not been studied. In the present study, we demonstrated that ethanol-activated CaMKII induced JNK1-dependent inflammasome activation. Previous report suggested that JNK was shown to be directly activated by CaMKII in rat brain astrocyte cells [58]. Indeed, we confirmed that the inhibition of CaMKII significantly decreased the level of JNK1 phosphorylation. Therefore, we confirmed that CaMKII is an upstream molecule of JNK1 that is important for ethanol-induced inflammasome activation. Previously, an increase in NLRP3 protein expression was thought to be important for activation of inflammasome. However, recent studies suggested that Ser 194 phosphorylation of NLRP3 was essential for the control of inflammasome activation and that JNK1 directly phosphorylates NLRP3, which stimulates the self-association and oligomerization of NLRP3 [25, 26]. In addition to NLRP3, phosphorylation of ASC is involved in the activation of inflammasome. Hara et al. demonstrated that JNK1-mediated phosphorylation of mouse ASC at Tyr 144 modulates inflammasome activation through ASC oligomerization [59]. Although we have not analyzed the direct association between JNK1 and NLRP3 or ASC because they were not our primary concern, our study was consistent with previous researchers’ reports in points of the JNK1-dependent inflammasome activation. Our results also demonstrated that JNK1 inhibition reduced the level of cleaved caspase-1 generated by inflammasome activation. Generally, inflammasome-activated caspase-1 induces pyroptosis in microglia [60]. However, our results showed that caspase-1 was involved in neuronal apoptosis as a regulator of parkin-mediated mitophagy.

Although many researchers have shown the protective role of mitophagy in oxidative stress, mitochondrial dysfunction, and apoptosis [61, 62], the regulation of mitophagy in an ethanol environment is still controversial. We found that ethanol-activated caspase-1 plays a key role in parkin-mediated mitophagy and neuronal apoptosis. Previous researchers have reported that ethanol administration induced parkin-mediated mitophagy, which was activated as a protective response to ethanol toxicity in the liver [63, 64]. Conversely, another study suggested that chronic ethanol consumption inhibited mitophagy through change in the expression of mitophagy-related regulator elements and induced hepatotoxicity [65]. In the present study, we confirmed that mitophagy was suppressed, even though PINK1 expression was increased, by ethanol. These results imply that PINK1/parkin-mediated mitophagy was insufficient or suppressed. A recent study reported that activated caspase-1 cleaved parkin to inhibit mitophagy and contribute to pyroptotic cell death in macrophage cell lines [35]. Caspase-1 has also been demonstrated to selectively cleave parkin at Asp 126 residue and inactivate the function of the ubiquitin-like domain in Chinese hamster ovary and human dopaminergic neuroblastoma SH-SY5Y cells [66, 67]. Interestingly, we found that ethanol treatment reduced the expression of parkin in the mitochondria and the expression was recovered by caspase-1 inhibition. This result suggests that caspase-1 activated by the ethanol-induced inflammasome inhibits parkin and that caspase-1 is important for mitophagy suppression. Although previous studies have shown that caspase-1 activated by inflammasome formation was associated with pyroptosis, which is a form of necrotic cell death [21], we propose that the ethanol-induced inflammasome stimulates neuronal apoptosis through mitophagy inhibition. The present study is the first to identify the detailed mechanism of CaMKII-mediated inflammasome activation and caspase-1-dependent mitophagy regulation in neuronal cells exposed to ethanol.

## Conclusions

In this study, we demonstrated that ethanol-induced calcium overload promoted excessive mitochondrial fission-mediated ROS production via CaMKII/Drp1 pathway and NLRP3 inflammasome-mediated mitophagy inhibition via CaMKII/JNK1 pathway, which induces neuronal cell apoptosis. The identification of the calcium signaling pathway regulating mitochondrial fission and the NLRP3 inflammasome activation will be useful in the development of therapeutic strategies for the treatment of ethanol-related neurodegenerative diseases.

## Supplementary information

**Additional file 1: Figure S1.** Effect of ethanol on NR1 accumulation on membrane. **A** Cells were incubated with EtOH for 12 h and immunostained with NR1 and Na^+^/K^+^-ATPase antibodies. Na^+^/K^+^-ATPase (green) and NR1 (red) were visualized with SRRF imaging system. Scale bars are 8 μm (magnification, × 1,000). Immunofluorescence images are representative.

**Additional file 2: Figure S2.** Effect of PBA on intracellular calcium concentration. **A** Cells were pretreated with PBA (2.5 mM) for 30 min, and then exposed to EtOH for 24 h. Cells were loaded with Fluo 3-AM (2 μM) for 30 min, and the amount of intracellular calcium was measured by using flow cytometry. Data are presented as a mean ± S.E.M. *n* = 3. NS is no significant difference between groups. *p < 0.05 versus control.

**Additional file 3: Figure S3.** Effect of ethanol on mRNA and protein expression of mitophagy regulator genes. **A** Cells were treated with EtOH (200 mM) for 24 h. mRNA expressions of *PINK1*, *BNIP3* and *NIX* were analyzed by quantitative real time PCR. Data were normalized by the ACTB mRNA expression level. Data are presented as a mean ± S.E.M. *n* = 3. **B** Cells were exposed to EtOH (200 mM) for 0–48 h. PINK1, BNIP3 and NIX were detected by western blot. β-Actin was used as a loading control. Data are presented as a mean ± S.E.M. *n* = 3. All blot images are representative. *p < 0.05 versus control.

**Additional file 4: Figure S4.** Effect of caspase-1 silencing on ethanol-reduced LC3-II expression in mitochondria. **A** Cells were transfected with *CASP1* siRNA or NT siRNA for 24 h prior to ethanol exposure for 48 h. LC3-II expression was measured by western blotting. β-Actin was used as a loading control. Data are presented as a mean ± S.E.M. *n* = 3. All blot images are representative. *p < 0.05 versus control, ^#^*p* < 0.05 versus EtOH.

**Additional file 5: Figure S5.** Effect of trehalose on ethanol-induced neuronal apoptosis. **A** Cells were pretreated with trehalose (2 μM) for 30 min prior to EtOH treatment for 72 h. Apoptotic cells were detected by annexin V/ PI staining. Data are presented as a mean ± S.E.M. *n* = 3. The data are representative. *p < 0.05 versus control, ^#^*p* < 0.05 versus EtOH.

**Additional file 6: Figure S6.** Law images in Fig. 1C-2E.

**Additional file 7: Figure S7.** Law images in Fig. 3A-F.

**Additional file 8: Figure S8.** Law images in Fig. 5A-6B.

**Additional file 9: Figure S9.** Law images in Fig. S1B-S2A.

**Additional file 10: Table S1.** Sequences of primers used for RT-PCR and real-time PCR

**Additional file 11: Table S2.** Sequences of siRNAs used for gene silencing

**Additional file 12: Table S3.** Information numbers of genes and proteins.

## Data Availability

All data generated and / or analyzed during the present study are available from the corresponding author on reasonable request.

## Abbreviations

EtOH: Ethanol
NMDAR: N-methyl-D-aspartate receptor
NLRP3: NOD-like receptor protein 3
CaMKII: Calmodulin-dependent protein kinase II
Drp1: Dynamin-related protein 1
CaM: Calmodulin
JNK1: c-Jun N-terminal protein kinase 1
ROS: Reactive oxygen species
PKA: Protein kinase A
CREB: cAMP-response-element-binding protein
TMRE: Tetramethylrhodamine ethyl ester
