# Supplementary material for: Ethanol-activated CaMKII signaling induces neuronal apoptosis through Drp1-mediated excessive mitochondrial fission and JNK1-dependent NLRP3 inflammasome activation
Source: Cell Commun Signal. 2020 Aug 12;18:123. doi: 10.1186/s12964-020-00572-3 (PMC7422600; doi:10.1186/s12964-020-00572-3)
Supplement: Supplementary file 2 — Additional file 1: Figure S1. Effect of ethanol on NR1 accumulation on membrane. A Cells were incubated with EtOH for 12 h and immunostained with NR1 and Na+/K+-ATPase antibodies. Na+/K+-ATPase (green) and NR1 (red) were visualized with SRRF imaging system. Scale bars are 8 μm (magnification, × 1,000). Immunofluorescence images are representative. [file 12964_2020_572_MOESM2_ESM.docx]

**A**


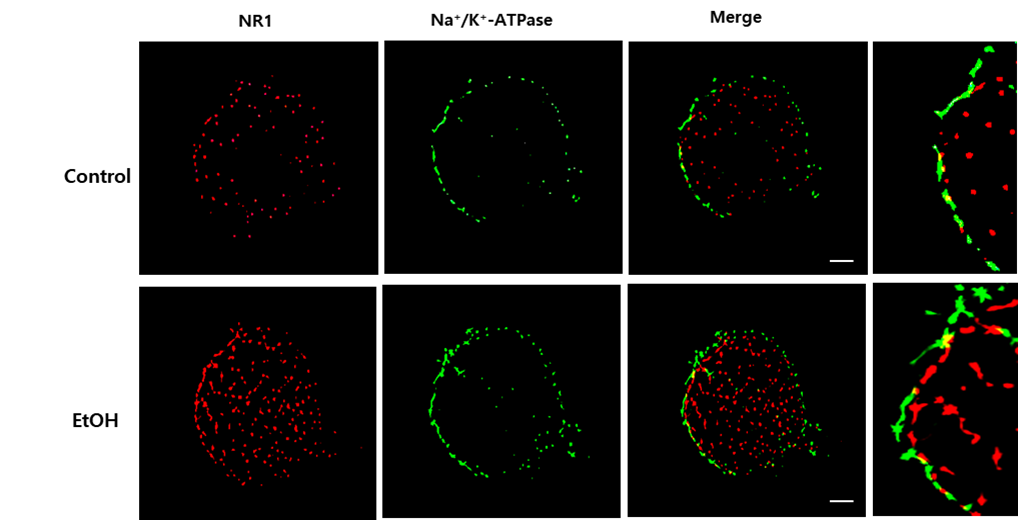


**Figure S1.** Effect of ethanol on NR1 accumulation on membrane. **A** Cells were incubated with EtOH for 12 h and immunostained with NR1 and Na^+^/K^+^-ATPase antibodies. Na^+^/K^+^-ATPase (green) and NR1 (red) were visualized with SRRF imaging system. Scale bars are 8 μm (magnification, × 1,000). Immunofluorescence images are representative.
