# Supplementary material for: Ethanol-activated CaMKII signaling induces neuronal apoptosis through Drp1-mediated excessive mitochondrial fission and JNK1-dependent NLRP3 inflammasome activation
Source: Cell Commun Signal. 2020 Aug 12;18:123. doi: 10.1186/s12964-020-00572-3 (PMC7422600; doi:10.1186/s12964-020-00572-3)
Supplement: Supplementary file 3 — Additional file 2: Figure S2. Effect of PBA on intracellular calcium concentration. A Cells were pretreated with PBA (2.5 mM) for 30 min, and then exposed to EtOH for 24 h. Cells were loaded with Fluo 3-AM (2 μM) for 30 min, and the amount of intracellular calcium was measured by using flow cytometry. Data are presented as a mean ± S.E.M. n = 3. NS is no significant difference between groups. *p < 0.05 versus control. [file 12964_2020_572_MOESM3_ESM.docx]

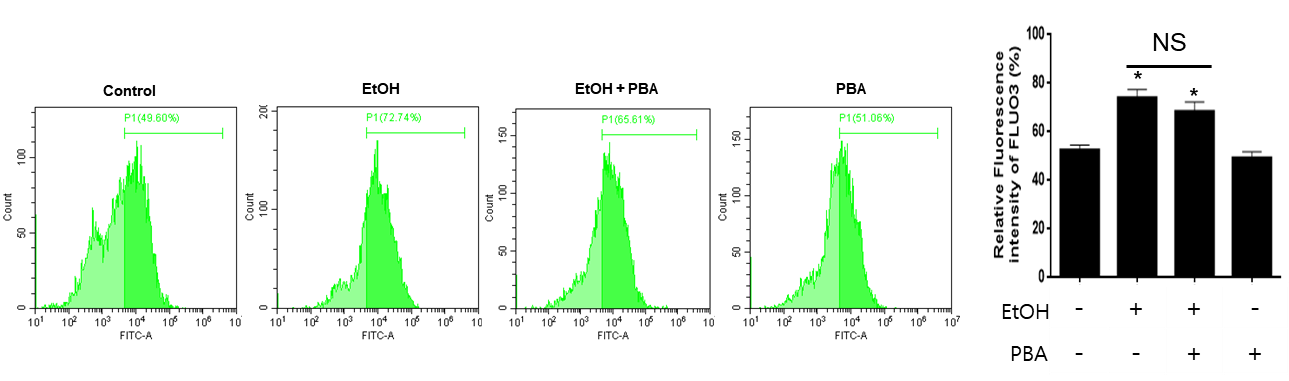


**Figure S2.** Effect of PBA on intracellular calcium concentration. **A** Cells were pretreated with PBA (2.5 mM) for 30 min, and then exposed to EtOH for 24 h. Cells were loaded with Fluo 3-AM (2 μM) for 30 min, and the amount of intracellular calcium was measured by using flow cytometry. Data are presented as a mean ± S.E.M. *n* = 3. NS is no significant difference between groups. **p* < 0.05 versus control.
