# Supplementary material for: Ethanol-activated CaMKII signaling induces neuronal apoptosis through Drp1-mediated excessive mitochondrial fission and JNK1-dependent NLRP3 inflammasome activation
Source: Cell Commun Signal. 2020 Aug 12;18:123. doi: 10.1186/s12964-020-00572-3 (PMC7422600; doi:10.1186/s12964-020-00572-3)
Supplement: Supplementary file 4 — Additional file 3: Figure S3. Effect of ethanol on mRNA and protein expression of mitophagy regulator genes. A Cells were treated with EtOH (200 mM) for 24 h. mRNA expressions of PINK1, BNIP3 and NIX were analyzed by quantitative real time PCR. Data were normalized by the ACTB mRNA expression level. Data are presented as a mean ± S.E.M. n = 3. B Cells were exposed to EtOH (200 mM) for 0–48 h. PINK1, BNIP3 and NIX were detected by western blot. β-Actin was used as a loading control. Data are presented as a mean ± S.E.M. n = 3. All blot images are representative. *p < 0.05 versus control. [file 12964_2020_572_MOESM4_ESM.docx]

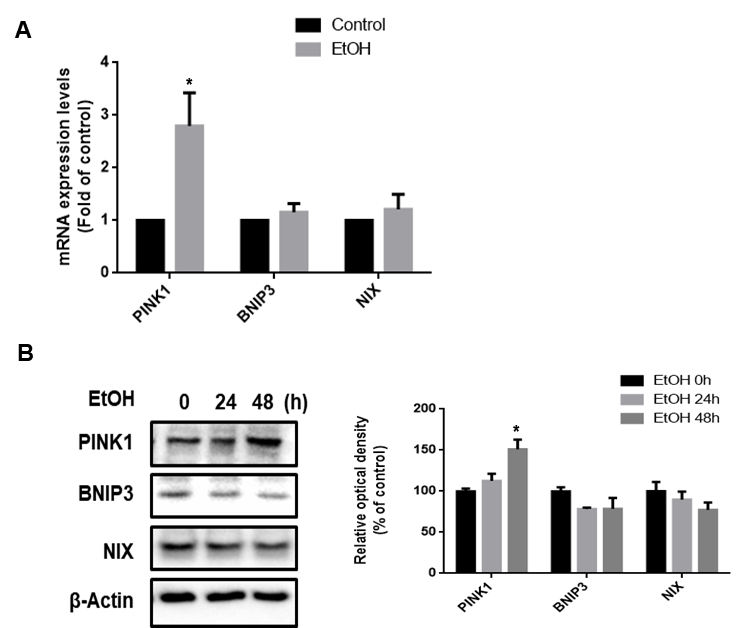


**Figure S3** Effect of ethanol on mRNA and protein expression of mitophagy regulator genes. **A** Cells were treated with EtOH (200 mM) for 24 h. mRNA expressions of *PINK1*, *BNIP3* and *NIX* were analyzed by quantitative real time PCR. Data were normalized by the ACTB mRNA expression level. Data are presented as a mean ± S.E.M. *n* = 3. **B** Cells were exposed to EtOH (200 mM) for 0-48 h. PINK1, BNIP3 and NIX were detected by western blot. β-Actin was used as a loading control. Data are presented as a mean ± S.E.M. *n* = 3. All blot images are representative. **p* < 0.05 versus control.
