# Supplementary material for: Ethanol-activated CaMKII signaling induces neuronal apoptosis through Drp1-mediated excessive mitochondrial fission and JNK1-dependent NLRP3 inflammasome activation
Source: Cell Commun Signal. 2020 Aug 12;18:123. doi: 10.1186/s12964-020-00572-3 (PMC7422600; doi:10.1186/s12964-020-00572-3)
Supplement: Supplementary file 5 — Additional file 4: Figure S4. Effect of caspase-1 silencing on ethanol-reduced LC3-II expression in mitochondria. A Cells were transfected with CASP1 siRNA or NT siRNA for 24 h prior to ethanol exposure for 48 h. LC3-II expression was measured by western blotting. β-Actin was used as a loading control. Data are presented as a mean ± S.E.M. n = 3. All blot images are representative. *p < 0.05 versus control, #p < 0.05 versus EtOH. [file 12964_2020_572_MOESM5_ESM.docx]

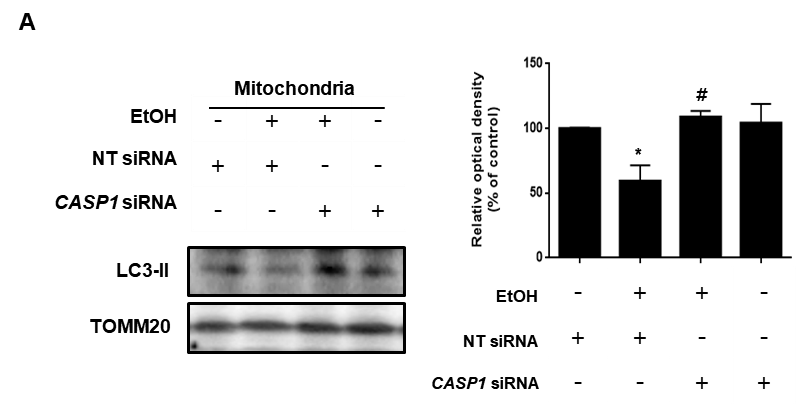


**Figure S4** Effect of caspase-1 silencing on ethanol-reduced LC3-II expression in mitochondria. **A** Cells were transfected with *CASP1* siRNA or NT siRNA for 24 h prior to ethanol exposure for 48 h. LC3-II expression was measured by western blotting. β-Actin was used as a loading control. Data are presented as a mean ± S.E.M. *n* = 3. All blot images are representative. **p* < 0.05 versus control, ^#^*p* < 0.05 versus EtOH.
