# Supplementary material for: Ethanol-activated CaMKII signaling induces neuronal apoptosis through Drp1-mediated excessive mitochondrial fission and JNK1-dependent NLRP3 inflammasome activation
Source: Cell Commun Signal. 2020 Aug 12;18:123. doi: 10.1186/s12964-020-00572-3 (PMC7422600; doi:10.1186/s12964-020-00572-3)
Supplement: Supplementary file 6 — Additional file 5: Figure S5. Effect of trehalose on ethanol-induced neuronal apoptosis. A Cells were pretreated with trehalose (2 μM) for 30 min prior to EtOH treatment for 72 h. Apoptotic cells were detected by annexin V/ PI staining. Data are presented as a mean ± S.E.M. n = 3. The data are representative. *p < 0.05 versus control, #p < 0.05 versus EtOH. [file 12964_2020_572_MOESM6_ESM.docx]

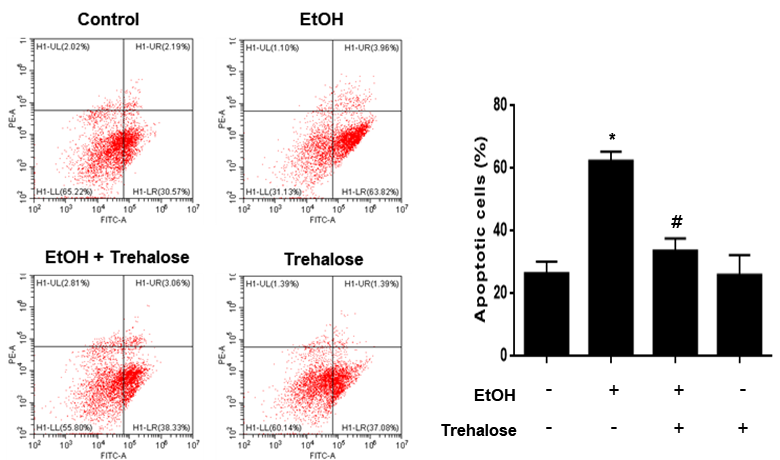


**Figure S5** Effect of trehalose on ethanol-induced neuronal apoptosis. **A** Cells were pretreated with trehalose (2 μM) for 30 min prior to EtOH treatment for 72 h. Apoptotic cells were detected by annexin V/ PI staining. Data are presented as a mean ± S.E.M. *n* = 3. The data are representative. **p* < 0.05 versus control, ^#^*p* < 0.05 versus EtOH.
