# Supplementary figures and images for: Ethanol-activated CaMKII signaling induces neuronal apoptosis through Drp1-mediated excessive mitochondrial fission and JNK1-dependent NLRP3 inflammasome activation
Source: Cell Commun Signal. 2020 Aug 12;18:123. doi: 10.1186/s12964-020-00572-3 (PMC7422600; doi:10.1186/s12964-020-00572-3)

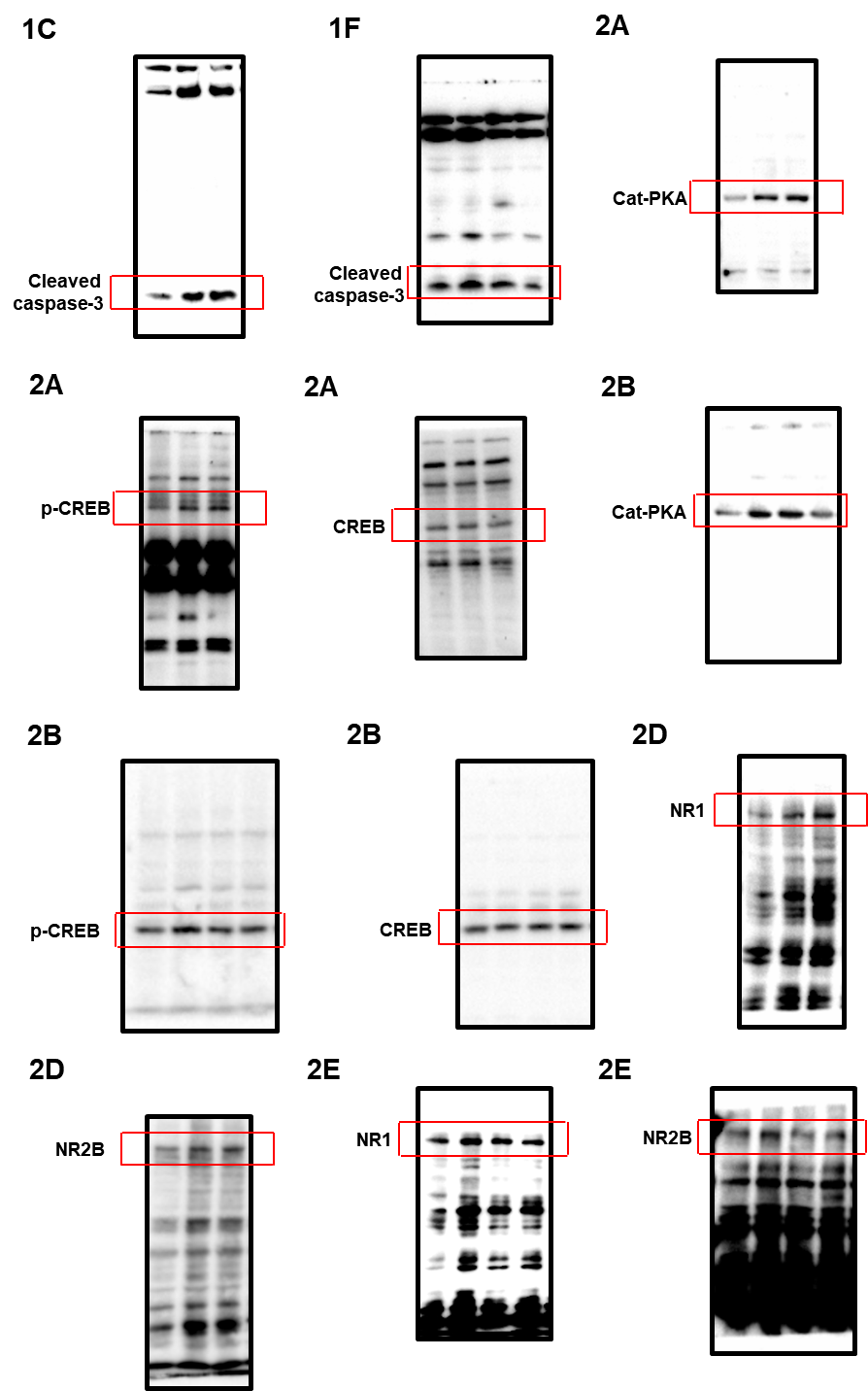


**Fig. S6** Law images in Fig.1C-2E.

Supplement: Supplementary file 7 — Additional file 6: Figure S6. Law images in Fig. 1C-2E. [file 12964_2020_572_MOESM7_ESM.docx]

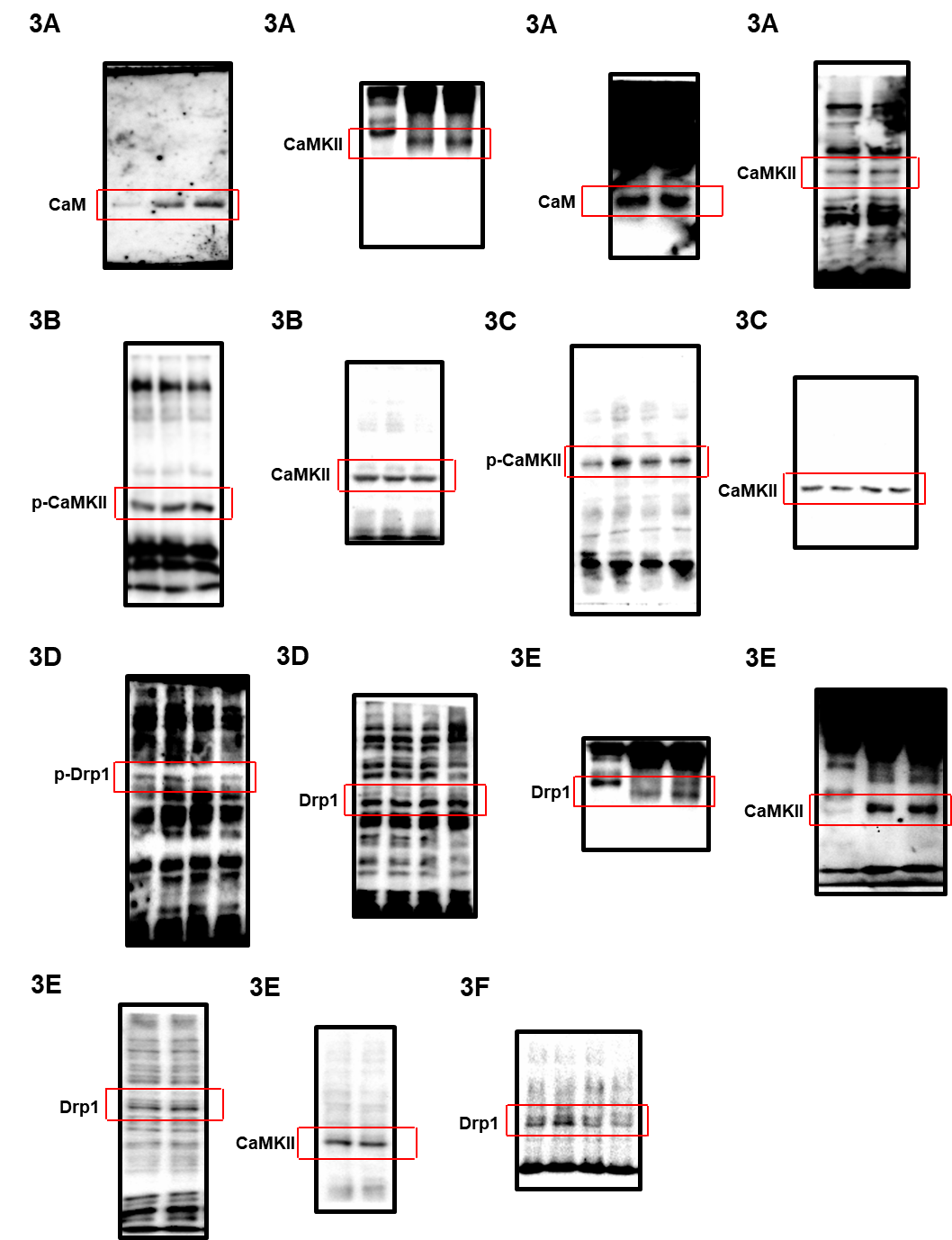


**Fig. S7** Law images in Fig. 3A-3F.

Supplement: Supplementary file 8 — Additional file 7: Figure S7. Law images in Fig. 3A-F. [file 12964_2020_572_MOESM8_ESM.docx]

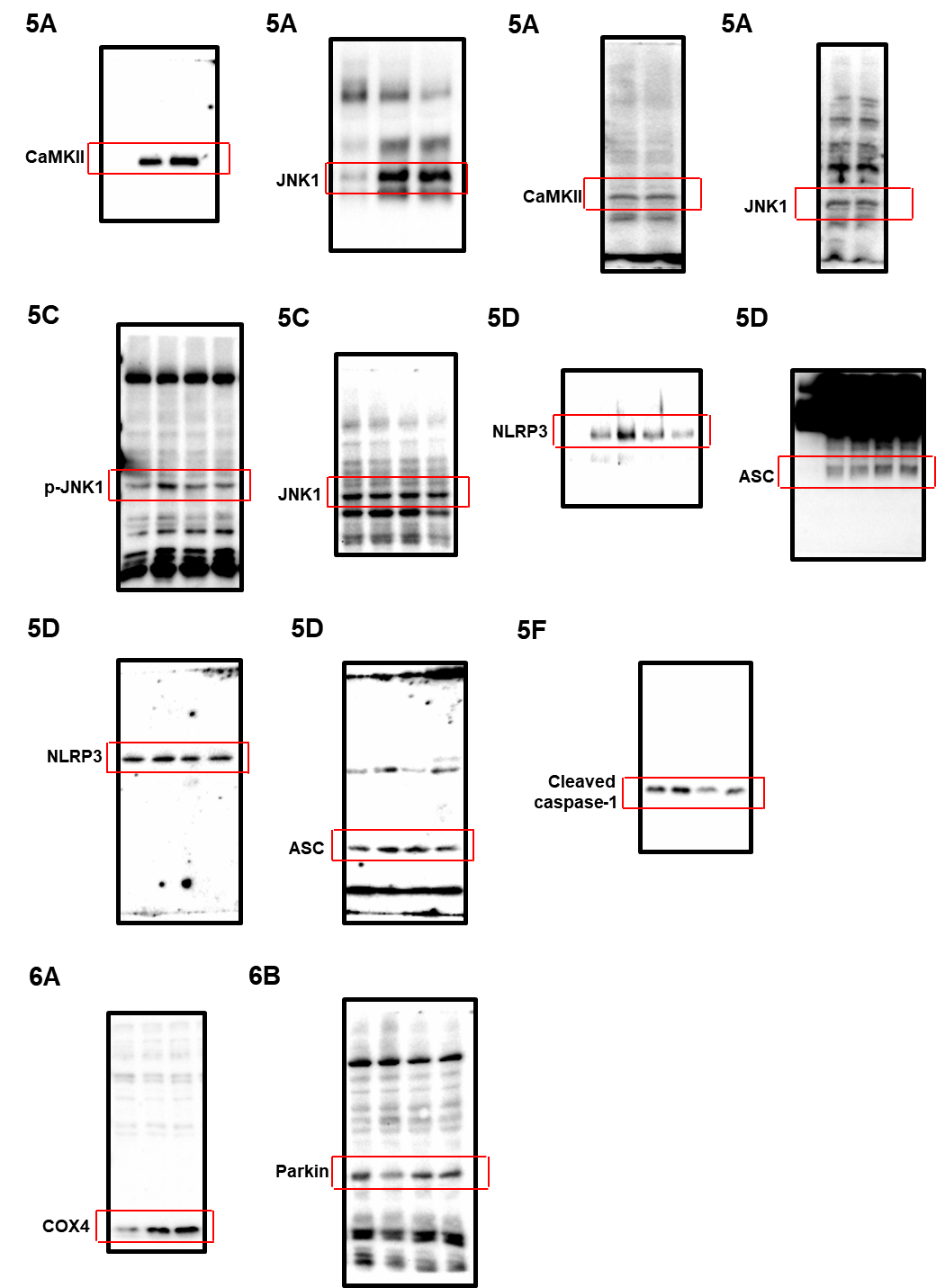


**Fig. S8** Law images in Fig. 5A-6B.

Supplement: Supplementary file 9 — Additional file 8: Figure S8. Law images in Fig. 5A-6B. [file 12964_2020_572_MOESM9_ESM.docx]

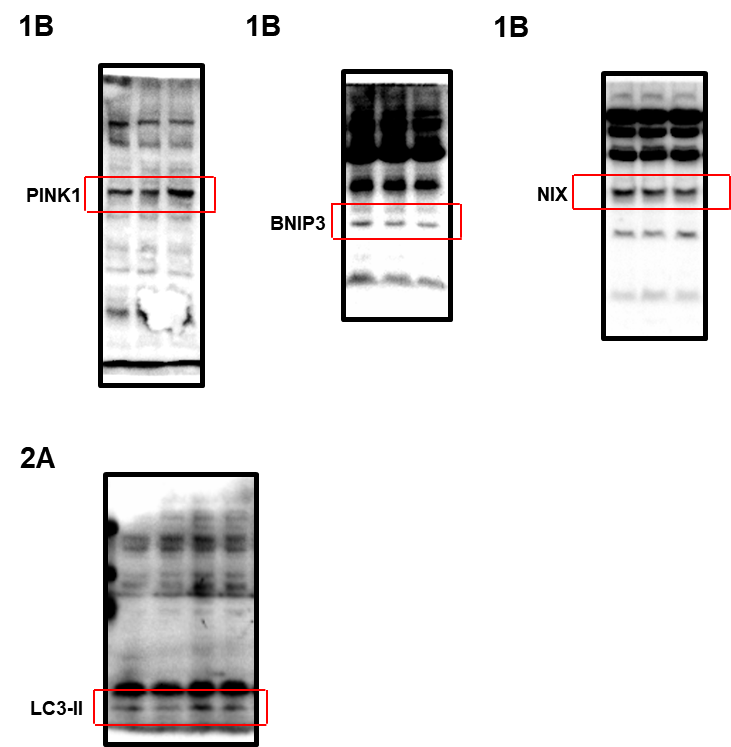


**Fig. S9** Law images in Fig. S1B-S2A.

Supplement: Supplementary file 10 — Additional file 9: Figure S9. Law images in Fig. S1B-S2A. [file 12964_2020_572_MOESM10_ESM.docx]
