# Supplementary material for: Ethanol-activated CaMKII signaling induces neuronal apoptosis through Drp1-mediated excessive mitochondrial fission and JNK1-dependent NLRP3 inflammasome activation
Source: Cell Commun Signal. 2020 Aug 12;18:123. doi: 10.1186/s12964-020-00572-3 (PMC7422600; doi:10.1186/s12964-020-00572-3)
Supplement: Supplementary file 11 — Additional file 10: Table S1. Sequences of primers used for RT-PCR and real-time PCR [file 12964_2020_572_MOESM11_ESM.docx]

**Table S1.** Sequences of primers used for RT-PCR and real-time PCR

| Gene | Identification | Sequence (5'-3') |
| --- | --- | --- |
| *GRIN1* | Forward | CTACCGCATACCCGTGCTG |
|  | Reverse | GCATCATCTCAAACCACACGC |
| *GRIN2A* | Forward | TGGCCTCACCGGGTATGATT |
|  | Reverse | CAATGCCGTCCCTCACTCTC |
| *GRIN2B* | Forward | TCTGACCGGAAGATCCAGGG |
|  | Reverse | TCCATGATGTTGAGCATTACGG |
| *GRIN2D* | Forward | GAGGAAAGGCCGTTTGTCATC |
|  | Reverse | TGTGGGTTCGGTTGAGCTG |
| *PINK1* | Forward | GCCTCATCGAGGAAAAACAGG |
|  | Reverse | GTCTCGTGTCCAACGGGTC |
| *BNIP3* | Forward | GCCATCGGATTGGGGATCTAT |
|  | Reverse | GCCACCCCAGGATCTAACAG |
| *NIX* | Forward | GGACTCGGCTTGTTGTGTTG |
|  | Reverse | TAGCTCCACCCAGGAACTGT |
| *ACTB* | Forward | AACCGCGAGAAGATGACC |
|  | Reverse | AGCAGCCGTGGCCATCTC |
