# Supplementary material for: Ethanol-activated CaMKII signaling induces neuronal apoptosis through Drp1-mediated excessive mitochondrial fission and JNK1-dependent NLRP3 inflammasome activation
Source: Cell Commun Signal. 2020 Aug 12;18:123. doi: 10.1186/s12964-020-00572-3 (PMC7422600; doi:10.1186/s12964-020-00572-3)
Supplement: Supplementary file 12 — Additional file 11: Table S2. Sequences of siRNAs used for gene silencing [file 12964_2020_572_MOESM12_ESM.docx]

**Table S2.** Sequences of siRNAs used for gene silencing

| Target gene | Sequences 5’-3’ | Manufacturer |
| --- | --- | --- |
| *CASP1* | GGAAGACUCAUUGAACAUA  GAUGGUAGAGCGCAGAUGC  CCGCAAGGUUCGAUUUUCA  GAGUGACUUUGACAAGAUG | Dharmacon |
| [*CREB1*](https://www.sciencedirect.com/topics/biochemistry-genetics-and-molecular-biology/creb1) | GAGAGAGGUCCGUCUAAUG  UAGUACAGCUGCCCAAUGG  CAACUCCAAUUUACCAAAC  GCCCAGCCAUCAGUUAUUC | Dharmacon |
| *JNK1* | GGAGCUCAAGGAAUAGUAU  AUACUAUUCCUUGAGCUCC | Cell Signaling Technology |
| Non-targeting | UAGCGACUAAACACAUCAA  UUGAUGUGUUUAGUCGCUA | Dharmacon |
