# Supplementary material for: Ethanol-activated CaMKII signaling induces neuronal apoptosis through Drp1-mediated excessive mitochondrial fission and JNK1-dependent NLRP3 inflammasome activation
Source: Cell Commun Signal. 2020 Aug 12;18:123. doi: 10.1186/s12964-020-00572-3 (PMC7422600; doi:10.1186/s12964-020-00572-3)
Supplement: Supplementary file 13 — Additional file 12: Table S3. Information numbers of genes and proteins. [file 12964_2020_572_MOESM13_ESM.docx]

**Table S3.** Information numbers of genes and proteins.

| Gene | Information number | Protein name | Information number |
| --- | --- | --- | --- |
| *GRIN1* | [NM_000832.7](https://www.ncbi.nlm.nih.gov/nuccore/NM_000832.7) | CaM | [P0DP23](https://www.uniprot.org/uniprot/P0DP23) |
| *GRIN2A* | [NM_000833.5](https://www.ncbi.nlm.nih.gov/nuccore/NM_000833.5) | CaMKII | [Q9UQM7](http://www.ncbi.nlm.nih.gov/sites/entrez?db=protein&cmd=search&term=Q9UQM7) |
| *GRIN2B* | [NM_000834.5](https://www.ncbi.nlm.nih.gov/nuccore/NM_000834.5) | CREB | [P16220](http://www.ncbi.nlm.nih.gov/sites/entrez?db=protein&cmd=search&term=P16220) |
| *GRIN2D* | [NM_000836.2](https://www.ncbi.nlm.nih.gov/nuccore/NM_000836.2) | JNK1 | [P45983](http://www.ncbi.nlm.nih.gov/sites/entrez?db=protein&cmd=search&term=P45983) |
| *PINK1* | [NM_032409.3](https://www.ncbi.nlm.nih.gov/nuccore/NM_032409.3) | BNIP3 | [Q12983](http://www.ncbi.nlm.nih.gov/sites/entrez?db=protein&cmd=search&term=Q12983) |
| *BNIP3* | [NM_004052.3](https://www.ncbi.nlm.nih.gov/nuccore/NM_004052.3) | NIX | [O60238](http://www.ncbi.nlm.nih.gov/sites/entrez?db=protein&cmd=search&term=O60238) |
| *NIX* | [NM_001330491.2](https://www.ncbi.nlm.nih.gov/nuccore/NM_001330491.2) | β-Actin | [P60709](http://www.ncbi.nlm.nih.gov/sites/entrez?db=protein&cmd=search&term=P60709) |
|  |  | Drp1 | [O00429](http://www.uniprot.org/uniprot/O00429) |
|  |  | PINK1 | [Q9BXM7](http://www.uniprot.org/uniprot/Q9BXM7) |
|  |  | Caspase-3 | [P42574](http://www.uniprot.org/uniprot/P42574) |
|  |  | TOMM20 | [Q15388](http://www.uniprot.org/uniprot/Q15388) |
|  |  | Parkin | [O60260](http://www.uniprot.org/uniprot/O60260) |
|  |  | NR2B | [Q13224](http://www.uniprot.org/uniprot/Q13224) |
|  |  | NR1 | [Q05586](http://www.uniprot.org/uniprot/Q05586) |
|  |  | COX4 | [P13073](http://www.uniprot.org/uniprot/P13073) |
|  |  | NLRP3 | [Q96P20](http://www.uniprot.org/uniprot/Q96P20) |
|  |  | Caspase-1 | [P29452](http://www.uniprot.org/uniprot/P29452) |
|  |  | LC3 | [Q9GZQ8](http://www.uniprot.org/uniprot/Q9GZQ8) |
